# Characterising how a single bout of exercise in people with myeloma affects clonal plasma cell and immune effector cell frequency in blood, and daratumumab efficacy *in vitro*

**DOI:** 10.1016/j.bbih.2024.100865

**Published:** 2024-09-19

**Authors:** Harrison D. Collier-Bain, Annabelle Emery, Frankie F. Brown, Adam J. Causer, Rebecca Oliver, Rachel Eddy, Shoji Leach, John Graby, Daniel Augustine, Sally Moore, Josephine Crowe, James Murray, James E. Turner, John P. Campbell

**Affiliations:** a Department for Health, University of Bath, United Kingdom; b School of Applied Sciences, Edinburgh Napier University, Edinburgh, United Kingdom; c Department of Haematology, Royal United Hospitals Bath NHS Foundation Trust, United Kingdom; d Department of Cardiology, Royal United Hospitals Bath NHS Foundation Trust, United Kingdom; e School of Sport, Exercise and Rehabilitation Sciences, University of Birmingham, Birmingham, United Kingdom; f School of Medical and Health Sciences, Edith Cowan University, Perth, Australia

**Keywords:** Myeloma_1_, Daratumumab_2_, Exercise_3_, ADCC_4_, NK-cells_5_, monocytes_5_, B-cells_6_

## Abstract

Multiple myeloma is a haematological cancer characterised by the accumulation of clonal plasma cells in the bone marrow and is commonly treated with daratumumab, an anti-CD38 monoclonal antibody immunotherapy. Daratumumab often fails to induce stringent complete responses, due in part to resistance to antibody-dependent cellular cytotoxicity (ADCC) exerted by natural killer (NK)-cells and monocytes. Exercise bouts undertaken by healthy people induce lymphocytosis in blood, including to NK-cells and B-cells, but the effects of exercise are unknown in myeloma patients. In addition, whether exercise mobilises plasma cells has not been adequately investigated, and as such the potential impact of exercise on daratumumab treatment is unclear. In this exploratory pilot study, *n* = 16 smouldering multiple myeloma participants enrolled and *n* = 9 completed the study which comprised a bout of cycling 15% above anaerobic threshold for ∼30-min, with blood samples collected pre-, immediately post-, and 30-min post-exercise. Peripheral blood mononuclear cells were isolated from blood samples and incubated with the RPMI-8226 plasmacytoma cell line, with or without the presence of daratumumab to determine specific lysis using a calcein-release assay. Daratumumab-mediated cell lysis increased from 18.8% to 23.2% pre- to post-exercise, respectively (*p* < 0.001), owing to an increased frequency of CD3^−^CD56^+^CD16^+^ NK-cells (+348%), HLA-DR^+^CD14^dim^CD16^+^ monocytes (+125%), and HLA-DR^+^CD14^+^CD32^+^ monocytes (+41%) in blood (*p* < 0.01). However, overall, total plasma cells (CD38^+^CD138^+^) nor clonal plasma cells (CD38^bright^CD138^+^CD45^−/dim^CD19^−^ with light-chain restriction) increased in blood (*p* > 0.05). Notably, we observed a 305% increase in NK-cells expressing CD38, the daratumumab target antigen, which might render NK-cells more susceptible to daratumumab-mediated fratricide – whereby NK-cells initiate ADCC against daratumumab-bound NK-cells. In conclusion, exercise modestly improved the efficacy of daratumumab-mediated ADCC *in vitro*. However, plasma cells were largely unchanged, and NK-cells expressing CD38 – the daratumumab target antigen – increased in blood. Future research should consider the optimal timings of exercise during daratumumab treatment in myeloma to avert exacerbation of daratumumab-mediated NK-cell lysis.

## 1 Introduction

Multiple myeloma is the third most common haematological cancer in the UK (HMRN, 2019), accounting for 2% of all cancers and 2% of all cancer related deaths (Cancer Research UK, 2017). Myeloma incidence increases with age, with approximately two-thirds of patients diagnosed aged 65-years or older (Smith et al., 2015), and the highest incidence rates per 100,000 people are between 85- to 89-years of age (Cancer Research UK, 2014). Myeloma is characterised by the accumulation of clonal plasma cells in the bone marrow (Kyle et al., 2003) and likely develops from asymptomatic precursor conditions known as monoclonal gammopathy of undetermined significance (MGUS) and smouldering multiple myeloma (SMM) (Kyle et al., 2002; Landgren et al., 2009). People with the asymptomatic precursors, SMM or MGUS, do not experience symptoms of myeloma such as end organ damage (Rajkumar et al., 2014) and therefore, are monitored without treatment until disease progression. Once diagnosed with symptomatic myeloma, younger and/or relatively more physically fit patients, as determined by a haematologist, receive a combination of drugs such as anti-CD38 monoclonal antibody (mAb) therapy (e.g., daratumumab) alongside bortezomib, thalidomide, and dexamethasone (D-VTD) to control and manage the disease prior to a haematopoietic stem cell transplant (Moreau et al., 2019). Despite considerable improvements in progression free survival with the addition of daratumumab into myeloma therapy regimens, patients are likely to suffer relapse, due to the persistence of tumour cells after therapy, known as minimal residual disease (MRD) (Moreau et al., 2019; Avet Loiseau et al., 2021; Paiva et al., 2008). Indeed, whilst daratumumab therapy combinations prior to a stem cell transplant achieves favourable overall response rates of 93% (Moreau et al., 2019), there is marked heterogeneity in responses. For example, only 29% of patients achieve stringent complete responses following daratumumab induction therapy and subsequent stem cell transplant (Moreau et al., 2019). This is thought to be due in part to failure of antibody-dependent cellular cytotoxicity (ADCC) – a mechanism-of-action of daratumumab exerted by NK-cells and by monocytes (Nijhof et al., 2015). Resistance to ADCC is principally attributed to NK-cell depletion and exhaustion, both in blood and the bone marrow, during daratumumab therapy (Verkleij et al., 2023), coupled with bone marrow stromal cells promoting myeloma cell survival by inhibiting ADCC (de Haart et al., 2016).

It is well established that a sufficiently intensive bout of exercise in healthy individuals induces leukocytosis in blood, comprising lymphocytes – such as NK-cells and B-cells – and monocytes (Campbell and Turner, 2018). These lymphocytes mobilised through exercise bouts – specifically NK-cells – demonstrate enhanced cytotoxicity per cell against haematological cancer cell lines *in vitro* (Bigley et al., 2014). Additionally, NK-cells and monocytes mobilised through exercise in cancer populations express CD16 on their surface (Collier-Bain et al., 2024a), which is a vital receptor for inducing daratumumab-mediated ADCC (Nijhof et al., 2015) and therefore, exercise presents as a potential adjuvant therapy to enhance the efficacy of daratumumab in myeloma. We have recently shown that exercise-induced lymphocytosis enhanced the efficacy of immunotherapy in people with chronic lymphocytic leukaemia (CLL) *ex vivo* via the mobilisation of effector (CD16^+^ NK-cells) and target (CLL) cells – including B-cells with a phenotype indicative of lymphoid origin – into blood (Collier-Bain et al., 2024a). However, how these findings translate to myeloma, a different blood cancer, is unclear. Unlike CLL, the bulk of clonal cells in myeloma reside in the bone marrow (Kyle et al., 2003), with only a small frequency of plasma cells circulating in blood (Sanoja-Flores et al., 2018; Termini et al., 2022). Whilst the migratory potential of plasma cells has been well described for some time (Kunkel and Butcher, 2003), and whilst it has been hypothesised that plasma cells might be mobilised by stress or exercise (Dhabhar et al., 2012), this has been difficult to investigate. Indeed, the frequency of plasma cells in the blood of healthy individuals is negligible, and thus it has not been adequately studied whether exercise increases plasma cell counts in blood in humans (Turner et al., 2016). Using EuroFlow next generation flow cytometry (Flores-Montero et al., 2017), circulating myeloma plasma cells were detected in 73–100% of SMM patients (Sanoja-Flores et al., 2018; Termini et al., 2022). To our knowledge, it has not been investigated whether exercise is able to increase the frequency of circulating plasma cells in myeloma patients. Mobilising plasma cells from the bone marrow may help to tackle ADCC resistance, and may offer a means of better detecting and monitoring MRD (Collier-Bain et al., 2023). Lastly, it is currently unknown whether exercise mobilises NK-cells or monocytes in myeloma patients, nor whether the mobilisation of these daratumumab effector cells has the potential to enhance anti-CD38 mAb therapy against circulating myeloma cells.

The aim of this exploratory clinical study was to characterise the effects of an individual bout of cycling at an intensity 15% above anaerobic threshold on the frequency of clonal plasma cells and immune cells in blood in people with SMM, and to determine whether any immunomodulatory changes arising in response to exercise altered daratumumab-mediated ADCC *in vitro* against a myeloma cell line.

## 2 Methods

### 2.1 Participants

**Fig. 1:**
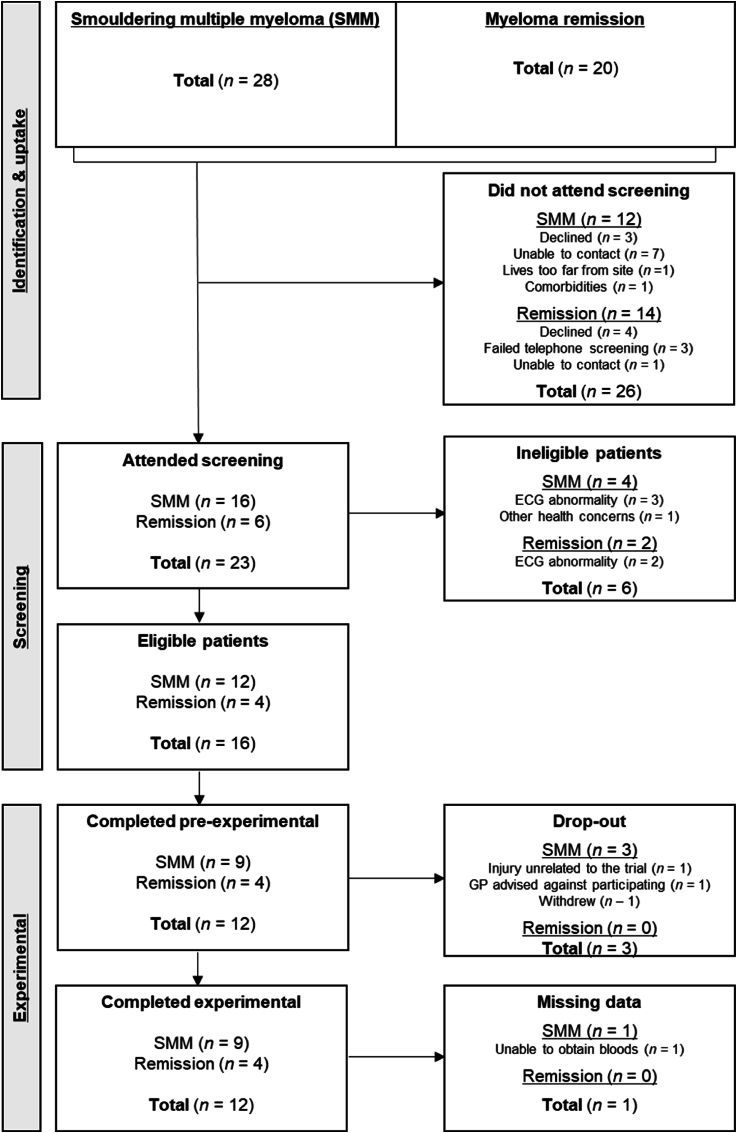
A CONSORT flow diagram of the recruitment and experimental completion of the study. SMM, smouldering multiple myeloma; GP, generational practitioner.

Patients with SMM (*n* = 16) were screened for eligibility (as discussed below) to perform strenuous exercise and all provided written informed consent, and *n* = 9 SMM patients subsequently completed all experimental procedures with no adverse events or serious adverse events occurring in this study. Eligible participant characteristics can be seen in Table 1. All participants were ≥18-years of age and were diagnosed as having SMM in-line with the 2014 International Myeloma Working Group (IMWG) criteria (Rajkumar et al., 2014). Participants were excluded if they were pregnant, reported an Eastern Cooperative Oncology Group (Oken et al., 1982) performance status >1 or were deemed unsafe to exercise via Physical Activity Readiness-Questionnaire (Thomas et al., 1992). Participants attended a screening visit and underwent a 12-lead electrocardiogram (ECG) which was reviewed by a cardiologist to confirm eligibility. Abnormal resting ECG resulted in *n* = 3 being excluded, with *n* = 1 being excluded due to other health concerns during in person screening, *n* = 1 who withdrew due to injury unrelated to the trial, *n* = 1 whose general practitioner advised against taking part, and *n* = 1 who withdraw after in-person screening, thus *n* = 9 completed the study (Fig. 1).

**Table 1:** Participant characteristics.

Variable	Smouldering multiple myeloma	Myeloma remission
Total participants (*n*)	9	4
Male/Female (*n*/*n*)	(4/5)	(3/1)
Ig light chain diagnosis (kappa/lambda)_b_	(7/2)	(3/1)
Ig heavy chain diagnosis (IgG/IgA)_b_	(8/1)	(4/0)
Age (years)	60 ± 9	68 ± 6
Height (cm)	173.6 ± 8.3	169.5 ± 10.1
Body mass (kg)	82.3 ± 25.7	75.6 ± 8.9
Body mass index (kg⋅m^−2^)	27.3 ± 8.3	26.4 ± 2.7
Body fat (%)_a_	31.8 ± 12.7	24.2 ± 8.7
Systolic blood pressure (mmHg)	134 ± 13	144 ± 9
Diastolic blood pressure (mmHg)	84 ± 5	92 ± 8
Erythrocytes ( × 10^12^/L)	3.99 ± 0.30	3.87 ± 1.11
Haemoglobin (g/L)	127 ± 12	132 ± 24
Haematocrit (L/L)	0.361 ± 0.024	0.381 ± 0.076
Leukocytes ( × 10^9^/L)	4.23 ± 1.45	3.45 ± 1.38
Lymphocytes ( × 10^9^/L)	1.44 ± 0.54	0.83 ± 0.32
Neutrophils ( × 10^9^/L)	2.30 ± 1.00	2.09 ± 1.28
Monocytes ( × 10^9^/L)	0.35 ± 0.07	0.38 ± 0.21
Anaerobic threshold (mL⋅kg^−1^⋅min^−1^)	15.2 ± 3.9	15.6 ± 2.3
Anaerobic threshold (W)	68 ± 31	65 ± 22

^a^Assessed via bioelectrical impedance Tanita scales, _b_Clinical diagnosis by immunofixation electrophoresis. Ig, immunoglobulin.

This study was primarily focussed on patients with SMM, as this patient group is treatment-naïve yet commonly has detectable clonal cells in blood (Sanoja-Flores et al., 2018; Termini et al., 2022). Myeloma patients in remission (MREM) were also recruited to preliminarily explore how a single bout of exercise affected immune cell frequency, including clonal plasma cells, in blood of patients in remission. Patients were deemed eligible if they had received a successful haematopoietic stem cell transplant as first-line treatment. Inclusion/exclusion criteria were as described for SMM. For this exploratory analysis, a total of *n* = 7 MREM patients provided written informed consent, and *n* = 4 completed all experimental procedures. Abnormal resting ECG resulted in *n* = 2 being excluded, and *n* = 1 who withdrew from the study due to illness unrelated to the trial (Fig. 1). Of the *n* = 4 MREM patients who completed all experimental procedures, *n* = 3 were on lenalidomide maintenance therapy following haematopoietic stem cell transplant, and *n* = 1 was on daratumumab maintenance therapy following haematopoietic stem cell transplant. This study was approved by the Health Research Authority and Health and Care Research Wales (21/EE/0202) and registered with an International Standard Randomised Controlled Trial Number (ISRCTN: 10197225).

### 2.2 Pre-experimental procedures

Participants arrived at the laboratory in the morning or the afternoon, having avoided strenuous exercise for 24-h prior to the visit and following a ≥4-h fast. Height was measured using a stadiometer (Seca, Birmingham, UK), body mass and fat percentage were measured using electronic scales with bioelectrical impedance analysis (Tanita Body Composition Analyser, SC-240, MA, Tokyo, Japan). Blood pressure measurements were taken supine, in triplicate, using an automated blood pressure monitor (OMRON, Kyoto, China) following ∼10-min of rest. Subsequently, anaerobic threshold was determined using an incremental sub-maximal ramp test on a Lode Excalibur cycle ergometer (Groningen, The Netherlands), as previously described (Collier-Bain et al., 2024a). Breath-by-breath gas exchange/ventilation (Carefusion, Vyntus CPX, CA, USA), heart rate via 12-lead ECG (Carefusion Vyntus ECG, CA, USA), and arterial oxygen saturation (SpO_2_) via pulse oximetry (Nonin PureSAT, MN, USA) were recorded continuously during exercise, whilst rating of perceived exertion (RPE) (6–20 Borg scale) (Borg, 1982) was recorded every minute and blood pressure was recorded pre- and post-incremental exercise. Pulmonary oxygen uptake ($\dot{\dot{V}}O_{2}$), carbon dioxide production ($\dot{\dot{V}}{\text{CO}}_{2}$), and ventilatory equivalents of O_2_ $\left({\dot{V}}_{E}/\dot{V}O_{2}\right)$ and CO_2_ (${\dot{V}}_{E}/\dot{\dot{V}}{\text{CO}}_{2}$) data were interpolated to 15-s averages. Anaerobic threshold was determined – independently by two researchers – using the V-slope method (Beaver et al., 1986) and confirmed through visual inspection of ${\dot{V}}_{E}/\dot{V}O_{2}$ and ${\dot{V}}_{E}/\dot{\dot{V}}{\text{CO}}_{2}$. Anaerobic threshold was reported in terms of $\dot{\dot{V}}O_{2}$ (mL⋅kg^−1^⋅min^−1^), power output (W), as a percentage of $\dot{\dot{V}}O_{2}$ (%) and as a percentage of age predicted maximum heart rate, using the following equation:

% age predicted maximum heart rate = (measured heart rate ÷ (220 – age in years)) × 100

### 2.3 Experimental procedures

After a minimum of 3-days, participants returned to the laboratory between 8:00–10:00 having avoided strenuous exercise and alcohol for 24-h, caffeine for ≥10-h, and following a ≥10-h fast. Body mass was reassessed, and participants were asked to rest in a supine position for ∼30-min prior to three blood pressure measurements the average of which was recorded. A 45 mL resting blood sample (pre-exercise) was then drawn from the antecubital vein via venepuncture. Participants then completed a 5-min warm-up on a cycle ergometer (Lode Excalibur, Groningen, The Netherlands) at 10% of the subsequent workload, followed by 30-min of cycling at a workload corresponding to 15% above their anaerobic threshold – considered a vigorous exercise intensity which has been previously shown to elicit a significant mobilisation of CD16^+^ NK-cells into blood and improve the efficacy of mAb immunotherapy (Collier-Bain et al., 2024a). A cadence of 60–80 revolutions per minute was maintained throughout and breath-by-breath gas exchange/ventilation, heart rate, and SpO_2_ were recorded continuously, whilst RPE was recorded every 5-min. Immediately following exercise cessation (within 3-min), another 45 mL blood sample (post-exercise) was drawn, with one final 45 mL blood sample drawn 30-min after exercise (30-min post-exercise) (all via venepuncture).

### 2.4 Sample processing

Blood samples were collected into sodium heparin (17 IU/mL), ethylenediaminetetraacetic-acid (EDTA, 1.8 mg/mL) and silica act clot activator treated vacutainers (Becton & Dickson, NJ, USA). Peripheral blood mononuclear cells (PBMCs) were isolated from heparinised blood, using SepMate™ tubes (StemCell Technologies, Vancouver, Canada) following manufacturer recommendations. PBMCs were cryopreserved at a concentration of 2–10 × 10^6^ cells/mL in freezing medium (heat-inactivated foetal calf serum [HI-FCS] + 10% [v/v] dimethyl sulfoxide [DMSO] [Invitrogen™, Thermo Fisher Scientific, Loughborough, UK]) in Mr. Frosty™ (Thermo Fisher Scientific, Loughborough, UK) at −80 °C for a minimum of 5-h and a maximum of 24-h and transferred to a −150 °C freezer for long-term storage.

EDTA-treated whole blood was analysed for blood lactate and blood glucose concentrations using rapid analysers (Lactate Plus Meter, Nova Biomedical, MA, USA and FreeStyle Optium Neo, Berkshire, UK, respectively) and then refrigerated (4 °C). EDTA-treated blood was then centrifuged (Heraeus Biofuge Primo R, Thermo Fisher Scientific, Loughborough, UK) within 2-h of collection at 2000×*g*, 4 °C for 15-min for the isolation of plasma. Silica-treated blood was allowed to clot for 60-min prior to centrifugation at 1300×*g*, 4 °C for 10-min for the isolation of serum. Both plasma and serum were immediately cryopreserved at −80 °C for long-term storage.

### 2.5 Whole blood counts

EDTA-treated blood was analysed in triplicate using an automated haematology analyser (Sysmex Kx-21N, Kobe, Japan) for leukocytes, erythrocytes, haemoglobin, haematocrit, and proportions/numbers of lymphocytes, monocytes, neutrophils within 2-h of collection.

### 2.6 Antibody-dependent cellular cytotoxicity (ADCC) assay

To analyse the effects of exercise on daratumumab-mediated ADCC in SMM, RPMI-8226 plasmacytoma cells (CD38^+^, myeloma; ECACC 87012702) were cultured in medium, containing glutamine enriched RPMI-1640 (Gibco™, MA, USA), supplemented with 10% (v/v) HI-FCS (Gibco™, MA, USA), 1% (v/v) penicillin/streptomycin (Thermo Fischer Scientific, Loughborough, UK), 1% (v/v) sodium pyruvate (Gibco™, MA, USA). RPMI-8226 cells were passaged into fresh medium every 2-days for 10-days before being frozen at −150 °C. RPMI-8226 cells were then thawed 7-days before being used in experimental assays and cultured under the conditions described above to ensure all target cells used in this study were equivalent. RPMI-8226 cells cultured for experiments were tested negative for mycoplasma (MycoAlert® PLUS Mycoplasma Detection Kit, Lonza, Slough, UK) following manufacturer instructions (data not shown).

RPMI-8226 cells (2 × 10^6^) were labelled with the membrane permeable molecule, calcein acetoxymethyl ester (calcein-AM) (Invitrogen™, Thermo Fisher Scientific, Loughborough, UK). Calcein-AM passively diffuses across target cell membrane where acetoxymethyl ester hydrolysis converts it into calcein, a green, fluorescent dye. Calcein is membrane-impermeable, therefore, the amount of calcein released is proportional to the amount of cell lysis. Labelled RPMI-8226 were washed three times by centrifugation at 500×*g*, 21 °C for 5-min (to remove excess calcein) and resuspended at a final concentration of 2 × 10^5^ cells/mL, achieving a 93 ± 5% viability of labelled RPMI-8226. Next, 5 × 10^3^ RPMI-8226 were seeded in round-bottom 96-well non-tissue culture treated plates (Falcon®, Corning, NY, USA) with 10 μg/mL of either anti-CD38 daratumumab (ADCC) or anti-HER2 herceptin (isotype control; antibody independent cellular cytotoxicity [AICC]) (Selleckchem, TX, USA) and opsonised for 30-min at 37 °C, 5% CO_2_. RPMI-8226 cells were also seeded in control wells, which were cultured with phosphate buffered saline (PBS; KCl 0.2 g/L, KH_2_PO_4_ 0.2 g/L, NaCl 8.0 g/L, Na_2_HPO_4_ 1.15 g/L; without CaCl_2_ and MgCl_2_ herein) (Sigma Aldrich, MI, USA) supplemented with 10% [v/v] of either HI-FCS, resting-plasma, or exercised-plasma to determine spontaneous lysis (negative control), and maximum lysis following treatment with 100 μL/well of 4% (v/v) Triton X-100 (positive control) (Invitrogen™, Thermo Fisher Scientific, Loughborough, UK), described below.

PBMCs from pre- and post-exercise were suspended in PBS at 2 × the concentration of blood they were isolated from and diluted 1:1 in a 96-well plate to achieve a final concentration equivalent to that observed in blood. To explore whether RPMI-8226 lysis was specific to the interactions between daratumumab bound to target cells and CD16/CD32 on the surface of NK-cells and monocytes, a fraction of PBMCs were treated with 50 μg/mL anti-CD16 (B73.1) and anti-CD32 (AT10) mAbs – used as blocking antibodies – (Invitrogen™, Thermo Fisher Scientific, Loughborough, UK) for 1-h at room-temperature. It should be noted here that two isoforms of CD16 exist (CD16a, CD16b) and three isoforms of CD32 exist (CD32a, CD32b, CD32c) where CD16a and CD32a are considered the primary receptors involving NK-cell and monocyte mediated ADCC, respectively. Nevertheless, CD16 on NK-cells is typically the CD16a isoform, with CD16b restricted to neutrophils (Ravetch and Perussia, 1989), and CD32a is expressed on the majority of monocytes, with dim expression of CD32b and CD32c (Zhao et al., 2019). Thus, the use of blocking antibodies which block the entire CD16 and CD32 receptor is sufficient to elucidate the involvement of NK-cells and monocytes in daratumumab-mediated killing. To investigate the influence of human plasma on cell lysis, wells were topped up with 10% of either HI-FCS, resting (R)-plasma, or exercised (Ex)-plasma. In another condition, whole blood collected from each time-point was added in 100 μL volumes to respective wells. PBS was then added to respective wells so that a 200 μL final volume was achieved following the addition of PBMCs or whole blood. The plate was then incubated for 2-h at 37 °C, 5% CO_2_.

Following incubation, 100 μL of 4% Triton X-100 was added to positive control wells and the plate was centrifuged at 100×*g* for 2-min at room-temperature. Subsequently 75 μL/well of acellular supernatant was transferred to a 96-well flat-bottom black plate (Corning™, Thermo Fischer Scientific, Loughborough, UK) and fluorescence measured (485 nm, 530 nm) using a Pherostar plate reader (BMG Labtech, Ortenberg, Germany) with the gain (based on positive controls) and optical height optimised for each plate. All conditions were seeded in triplicate wells and relative fluorescent units were converted to a percentage of specific lysis using the following equation:

%SpecificLysis=((Sample–Spontaneous)/(TritonX−100–Spontaneous))x100

### 2.7 Immunophenotyping

Sodium heparin-treated whole blood (300 μL for tubes used to identify plasma cells, and 100 μL for tubes used to identify monocytes and T-cells) were labelled with surface staining antibody-fluorochrome cocktails for 30-min (discussed later) at room temperature and subsequently treated with 1 × FACS Lysing Solution (BD Biosciences, Wokingham, UK) for 10-min at room temperature. Cells were washed twice in PBS before monocyte and T-cell tubes were resuspended in a final volume of 250 μL PBS. Following washes, plasma cell tubes were treated with a Fix/Perm solution (Cytofix-Cytoperm™, BD Biosciences, Wokingham, UK) for 20-min at room temperature followed by a 10-min incubation at room temperature with a perm/wash buffer (Cytofix-Cytoperm™, BD Biosciences, Wokingham, UK) and subsequently centrifuged at 500×*g* for 5-min, 21 °C. Cells were resuspended in perm/wash buffer and labelled with intracellular antibodies (e.g., Ig-kappa [Igκ] and Ig-lambda [Igλ]) for 30-min at room temperature. Cells were then washed once in perm/wash buffer and subsequently resuspended in a final volume of 500 μL PBS.

An additional panel was conducted to enumerate NK-cell subsets capable of, and susceptible to, daratumumab-mediated ADCC. Thawed PBMCs were washed twice by centrifugation at 500×*g* for 5-min, 21 °C and resuspended in PBS at a concentration of 0.5–1 × 10^7^ cells/mL. PBMCs were then seeded at a concentration of 0.5–1 × 10^6^ cells in 5 mL round-bottom polystyrene test tubes (Falcon®, Corning, NY, USA). Cells were treated with a fixable viability stain 510 (FVS510, BV510), following manufacturer instructions (BD Biosciences, Wokingham, UK) for 15-min at room temperature, washed by centrifugation at 500×*g* for 5-min, 21 °C and resuspended in 100 μL of MACS buffer (PBS, 10% [v/v] HI-FCS, and 2 mM EDTA [Invitrogen™, Thermo Fisher Scientific, Loughborough, UK]). Subsequently, cells from each time point were stained with surface staining antibody-fluorochrome cocktails (discussed below) for 30-min at room temperature followed by a final wash at 500×*g* for 5-min, 21 °C, before being resuspended in a final volume of 250 μL MACS buffer.

All antibodies were pre-titrated to ensure optimal fluorescent staining (data not shown) and both unstained cells from each time point, and single stained tubes containing anti-mouse positive (Igκ) and negative control compensation particles (BD Biosciences, Wokingham, UK) were used in each assay to correct for spectral overlap. Data were analysed using FlowJo (Version 10.9, BD Biosciences, Wokingham, UK) and presented as cells/μL – calculated using total leukocyte frequency from whole blood counts.

#### 2.7.1 Plasma cell identification

**Fig. 2:**
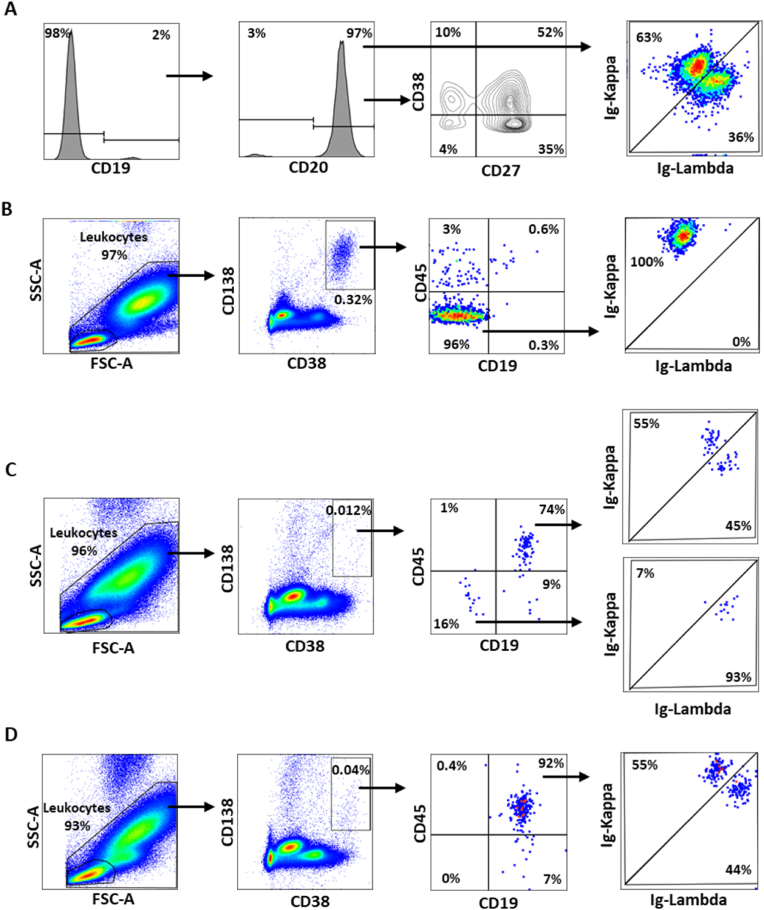
Representative gating strategies for plasma cells and B-cells. **A)** Displays normal B-cells in a SMM participant. CD19^+^ B-cells were identified in the lymphocyte population using a histogram, and further gated as CD20^+^. Within the CD19^+^CD20^+^ B-cell population, B-cell subsets were identified by plotting CD38 against CD27, and confirmed as polyclonal through an Ig-Kappa × Ig-Lambda plot. **B)** Displays a SMM participant with greater plasma cell burden. Following the exclusion of debris and doublets (not shown) leukocytes were identified in a SSC-A × FSC-A plot. Plasma cells were then identified as CD38^bright^CD138^+^ using a generous box gate. Clonal plasma cells were then identified as CD45^−/dim^CD19^−^ and confirmed through light chain restriction. **C)** Displays a SMM participant with few plasma cells, comprising both clonal plasma cells and polyclonal plasma cells – identified as CD45^+^CD19^+^ and with polyclonal light chains. **D)** Displays a MREM participant on lenalidomide maintenance with detectable plasma cells, comprising only polyclonal plasma cells. SSC-A, side scatter-area; FSC-A, forward scatter-area; SMM, smouldering multiple myeloma; MREM, myeloma remission.

Plasma cell populations were immunophenotyped using the following mAbs: anti-CD138-BV421 (MI15), anti-CD38-BB515 (HIT2), anti-CD45-BV510 (HI30), anti-CD20-BV605 (2H7), anti-CD56-PE (B159), anti-CD19-PE-Cy7 (HIB19), anti-CD117-APC (104D2), anti-CD81-AF700 (JS-81), anti-Igκ-PerCP-Cy5.5 (G20-193), anti-Igλ-APC-H7 (1-155-2) (BD Biosciences, Wokingham, UK), anti-CD27-PE-Cy5.5 (LPFS2/1611) (Novus Biologicals, Abingdon, UK). Briefly, plasma cells were identified in total leukocytes by strong CD38 co-expressed with strong CD138 expression and distinguished from polyclonal plasma cells by low/no expression of CD19 and CD45 and confirmed by light chain restriction. Representative gating strategies informed by previous research (Gonsalves et al., 2017) can be seen in Fig. 2.

#### 2.7.2 Natural killer (NK)-cell identification

**Fig. 3:**
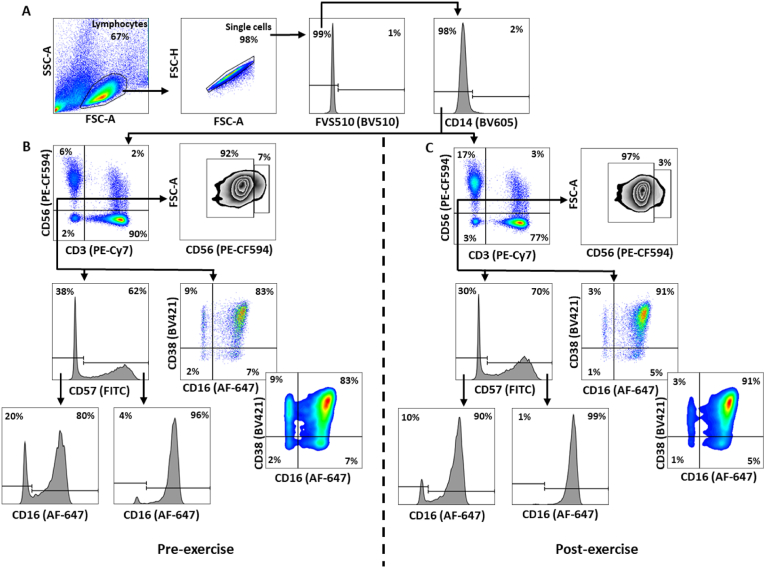
Representative gating strategy for NK-cells. **A)** Lymphocytes were identified in a SSC-A × FSC-A plot prior to the removal of doublets in a FSC-H × FSC-A plot. Next, a fixable viability stain (FVS) was used to remove non-viable cells prior the removal of monocytes using a histogram. **B)** In a pre-exercise sample, CD56 was plotted against CD3 to identify total CD3^−^CD56^+^ NK-cells. In the CD3^−^CD56^+^ NK-cell population: CD56^dim^ and CD56^bright^ NK-cells were identified in a FSC-A × CD56 plot; NK-cells capable of, and susceptible daratumumab-mediated ADCC were identified in a CD38 × CD16 plot; and CD57^−^ and CD57^+^ NK-cells were identified using a histogram. CD3^−^CD56^+^CD57^−^ and CD3^−^CD56^+^CD57^+^ NK-cells were further gated to identify CD16 sub-populations. **C)** Represents the same gating strategy as ‘**B)**’ but from a post-exercise sample. SSC-A, side scatter-area; FSC-A, forward scatter-area; FSC-H, forward scatter-height; SMM, smouldering multiple myeloma.

NK-cell populations were immunophenotyped via direct immunofluorescent antibody staining procedure of thawed PBMCs using the following antibodies: anti-CD3-PE-Cy7 (UCHT1), anti-CD56-PECF594 (NCAM16.2), anti-CD14-BV605 (M5E2), anti-CD38-BV421 (HIT2), anti-CD57-FITC (NK-1) (BD Biosciences, Wokingham, UK), and anti-CD16-AF647 (1001049) (R&D Systems, Abingdon, UK). Representative gating strategies can be seen in Fig. 3.

#### 2.7.3 Monocyte identification

Monocyte populations were immunophenotyped via direct immunofluorescent antibody staining procedure of whole blood using the following antibodies: anti-CD3-APC-H7 ((SK7), anti-CD14-PE-Cy5.5 (Tuk4), anti-CD16-BV510 (3G8), anti-CD32-PE (FLI8.26), anti-CD33-BV421 (P67.6), and anti-HLA-DR-AF700 (G46-6) (BD Biosciences, Wokingham, UK). This panel of antibodies was also used to identify cells with a myeloid-derived suppressor cell (MDSC)-like phenotype (HLA-DR^−^CD33^+^) (Verschoor et al., 2013; Gorgun et al., 2013). Representative gating strategies can be seen in Supplementary Fig. 1.

#### 2.7.4 T-cell identification

T-cell populations were immunophenotyped via direct immunofluorescent antibody staining procedure of whole blood using the following antibodies: anti-CD3-APC-H7 (SK7), anti-CD4-PE-Cy7 (SK3), anti-CD8-AF700 (RPA-T8), anti-PD-1-BB700 (EH12.1), anti-CD57-FITC (NK-1), anti-CTLA4-PE-Cy5 (BNI3) (BD Biosciences, Wokingham, UK), anti-CD28-APC (CD28.2) (Invitrogen™, Thermo Fisher Scientific, Loughborough, UK). Representative gating strategies can be seen in Supplementary Fig. 2.

### 2.8 Statistical analysis

Statistical analyses were conducted using SPSS (IBM SPSS Statistics Version 28, IL, USA). Data are presented as mean ± SD unless otherwise stated. One-way repeated measures analysis of variance (ANOVA) were performed to determine main effects of time (pre-, post-, 30-min post-exercise) for immune cell populations – including NK-cells, monocytes, B-cells, and T-cells. Paired sample t-tests or Wilcoxon signed-rank tests (if non-parametric distribution was observed following a Shapiro-Wilk test) were used to analyse differences pre- to post-exercise for PBMC AICC, ADCC, and ADCC + anti-CD16/32 and to determine differences between conditions cultured with HI-FCS and time-point matched autologous plasma. For whole blood ADCC, one-way repeated measures ANOVA was used to determine differences pre-, post-, and 30-min post-exercise in AICC and ADCC. Significant effects from ANOVA were subjected to *post hoc* comparisons with Bonferroni corrections to locate significant changes, as reported in Table/Figure legends. Physiological responses to exercise were analysed via one-way repeated measures ANOVA. Effect sizes were calculated within the statistical tests described above. Effect sizes for t-tests are Cohen's *d* with effect sizes determined small (*d* = 0.2), medium (*d* = 0.5), or large (*d* = 0.8). Effect sizes for ANOVA are partial eta squared (ηp^2^), with the effect sizes determined small (ηp^2^ = 0.01), medium (ηp^2^ = 0.06), or large (ηp^2^ = 0.14) (Lakens, 2013). The level of significance was set at *p* ≤ 0.05.

## 3 Results

### 3.1 Characteristics of cycling exercise

All participants completed a bout of cycling at an intensity corresponding to 15% above anaerobic threshold for 30-min with *n* = 1 SMM patient ceasing exercise at 16-min and 15-s due to volitional exhaustion. Physiological responses including, $\dot{\dot{V}}O_{2}$ (mL⋅kg^−1^⋅min^−1^), relative $\dot{\dot{V}}O_{2}$ as a percentage of anaerobic threshold (%), $\dot{\dot{V}}{\text{CO}}_{2}$ (mL⋅kg^−1^⋅min^−1^), $\dot{\dot{V}}E$ (L⋅min^−1^), respiratory exchange ratio, heart rate (bpm), heart rate as a percentage of age-predicted maximum (%), and RPE (6–20 Borg scale) averaged into two, 15-min segments are displayed in Table 2. Participants cycled above anaerobic threshold throughout the exercise trial, confirmed by $\dot{\dot{V}}O_{2}$ as a percentage of anaerobic threshold (%), which significantly increased from the warm-up to 15-min (107 ± 16%; *p* < 0.001), and 30-min (112 ± 16%; *p* < 0.001). Additionally, blood lactate significantly increased pre- to post-exercise by 114 ± 70% (*p* = 0.007) (Table 3). The effects of exercise on total leukocytes, lymphocytes, monocytes, and neutrophils obtained from whole blood counts are displayed in Table 3. Due to an unavailable blood sample within 3-min after completion of the exercise bout, *n* = 1 SMM data were excluded from subsequent analysis.

**Table 2:** Characteristics of cycling above anaerobic threshold with main effect from repeated measures ANOVA. Data are mean ± SD.

Variable	Warm-up	15-min	30-min	Main effect of time
*Smouldering multiple myeloma (n = 9)*				
$\dot{\dot{V}}O_{2}$ (mL⋅kg^−1^⋅min^−1^)	8.7 ± 1.7	16.3 ± 5.2^b^	17.3 ± 6.2^b^	*F*_(1.07,8.57)_ = 23.41, *p* < 0.001, ηp^2^ = 0.75
$\dot{\dot{V}}O_{2}$ (% anaerobic threshold)	59 ± 12	107 ± 16^c^	112 ± 16^c^	*F*_(1.26,10.05)_ = 58.87, *p* < 0.001, ηp^2^ = 0.88
$\dot{\dot{V}}{\text{CO}}_{2}$ (mL⋅kg^−1^⋅min^−1^)	6.7 ± 1.2	14.6 ± 5.0^b^	15.3 ± 5.8^b^	*F*_(1.10,8.34)_ = 25.83, *p* < 0.001, ηp^2^ = 0.76
$\dot{\dot{V}}E$ (L⋅min^−1^)	19.7 ± 4.8	39.2 ± 12.5^b^	42.9 ± 13.6^b^,^d^	*F*_(1.09,8.72)_ = 30.56, *p* < 0.001, ηp^2^ = 0.79
Respiratory exchange ratio	0.77 ± 0.03	0.89 ± 0.04^c^	0.88 ± 0.03^c^	*F*_(2,16)_ = 40.15, *p* < 0.001, ηp^2^ = 0.83
Heart rate (bpm)	79 ± 8	108 ± 20^b^	118 ± 26^c^,^f^	*F*_(1.05,8.39)_ = 39.78, *p* < 0.001, ηp^2^ = 0.83
Heart rate (% age predicted max)	49 ± 2	67 ± 11^c^	66 ± 18^b^	*F*_(1.13,9.03)_ = 20.00, *p* = 0.001, ηp^2^ = 0.71
Rating of perceived exertion	8 ± 2	12 ± 1^c^	13 ± 2^c^,^e^	*F*_(2,16)_ = 67.92, *p* < 0.001, ηp^2^ = 0.90
*Myeloma remission (n = 4)*				
$\dot{\dot{V}}O_{2}$ (mL⋅kg^−1^⋅min^−1^)	9.6 ± 0.7	18.4 ± 3.5^a^	19.5 ± 4.1^a^	*F*_(2,6)_ = 29.64, *p* < 0.001, ηp^2^ = 0.91
$\dot{\dot{V}}O_{2}$ (% anaerobic threshold)	62 ± 7	117 ± 6^b^	124 ± 8^a^	*F*_(2,6)_ = 73.96, *p* < 0.001, ηp^2^ = 0.96
$\dot{\dot{V}}{\text{CO}}_{2}$ (mL⋅kg^−1^⋅min^−1^)	7.3 ± 0.5	16.4 ± 3.1^a^	17.1 ± 3.5^a^	*F*_(2,6)_ = 32.63, *p* < 0.001, ηp^2^ = 0.92
$\dot{\dot{V}}E$ (L⋅min^−1^)	21.0 ± 1.2	43.9 ± 10.4^a^	47.6 ± 10.4^a^,^d^	*F*_(2,6)_ = 26.51, *p* = 0.001, ηp^2^ = 0.90
Respiratory exchange ratio	0.76 ± 0.03	0.90 ± 0.04^a^	0.88 ± 0.03^b^	*F*_(2,6)_ = 62.09, *p* < 0.001, ηp^2^ = 0.95
Heart rate (bpm)	78 ± 12	106 ± 15	119 ± 17^d^	*F*_(1.03,3.08)_ = 15.18, *p* = 0.029, ηp^2^ = 0.84
Heart rate (% age predicted max)	51 ± 7	69 ± 8	76 ± 10	*F*_(2,6)_ = 13.23, *p* = 0.006, ηp^2^ = 0.82
Rating of perceived exertion	8 ± 1	12 ± 1∗	13 ± 0.4^a^	*F*_(2,6)_ = 29.9, *p* < 0.001, ηp^2^ = 0.91

ANOVA, analysis of variance; bpm, beats per minute.

a indicates a significant difference from ‘warm-up’ at *p* < 0.05.

b indicates a significant difference from ‘warm-up’ at *p* < 0.01.

c indicates a significant difference from ‘warm-up’ at *p* < 0.001.

d indicates a significant difference from ’15-min’ at *p* < 0.05.

e indicates a significant difference from ’15-min’ at *p* < 0.01.

f indicates a significant difference from ‘15-min’ and *p* < 0.001.

**Table 3:** Haemodynamic variables pre-exercise, post-exercise, and 30-min post-exercise with percentage change (%Δ) pre-to post-exercise and main effect from one-way repeated measures ANOVA in smouldering multiple myeloma. Data are mean ± SD, *n* = 8.

Variable	Pre-exercise	Post-exercise	30-min post-exercise	%Δ Pre-Post	Main effect of time
Leukocytes ( × 10^9^/L)	4.29 ± 1.54	6.23 ± 1.86^b^	4.24 ± 1.50^d^	49 ± 23	*F*_(1.06,7.43)_ = 48.38, *p* < 0.001, ηp^2^ = 0.87
Lymphocytes ( × 10^9^/L)	1.43 ± 0.57	2.34 ± 0.85^a^	1.42 ± 0.63^c^	68 ± 32	*F*_(1.04,7.31)_ = 25.13, *p* = 0.001, ηp^2^ = 0.78
Monocytes ( × 10^9^/L)	0.35 ± 0.08	0.53 ± 0.13^a^	0.35 ± 0.08^c^	48 ± 22	*F*_(2,14)_ = 23.48, *p* < 0.001, ηp^2^ = 0.77
Neutrophils ( × 10^9^/L)	2.36 ± 1.05	3.19 ± 1.22^b^	2.36 ± 0.93^d^	39 ± 18	*F*_(2,14)_ = 44.86, *p* < 0.001, ηp^2^ = 0.87
Erythrocytes ( × 10^12^/L)	4.00 ± 0.32	4.22 ± 0.32	3.87 ± 0.31^c^	6 ± 7	*F*_(2,14)_ = 9.23, *p* = 0.003, ηp^2^ = 0.57
Blood lactate (mmol/L)	1.1 ± 0.4	2.3 ± 0.8^a^	1.3 ± 0.5^c^	114 ± 70	*F*_(1.20,8.36)_ = 21.40, *p* = 0.001, ηp^2^ = 0.75
Blood glucose (mmol/L)	5.6 ± 0.6	5.7 ± 0.7	5.5 ± 0.8	2 ± 12	*F*_(2,14)_ = 0.50, *p* = 0.62, ηp^2^ = 0.07

a indicates a significant difference from pre-exercise at *p* < 0.01.

b indicates a significant difference from pre-exercise at *p* < 0.001.

c indicates a significant difference from post-exercise at *p* < 0.01.

d indicates a significant difference from post-exercise at *p* < 0.001.

### 3.2 Plasma cell and B-cell mobilisation during cycling exercise

Table 4 displays summary data and statistical results for the effects of exercise on plasma cells and B-cells. Upon investigating plasma cells, we found no significant effects of time for total (CD38^bright^CD138^+^) plasma cells, clonal plasma cells (CD38^bright^CD138^+^CD45^−/dim^CD19^−^ with light-chain restriction), or polyclonal plasma cells (CD38^bright^CD138^+^CD45^+^CD19^+^). We also explored whether cycling 15% above anaerobic threshold could mobilise clonal plasma cells into the blood in a subset of MREM patients. There were no clonal plasma cells detected in any MREM patients pre-exercise, with *n* = 1 having detectable clonal plasma cells post-exercise (0.0065 clonal plasma cells/μL), and *n* = 1 having detectable clonal plasma cells 30-min post-exercise (0.0071 clonal plasma cells/μL) (Supplementary Table 1).

**Table 4:** Plasma cells and B-cells pre-exercise, post-exercise, and 30-min post-exercise in participants with smouldering multiple myeloma, with percentage change (%Δ) pre-to post-exercise and main effect of time from repeated measures ANOVA. Data are mean ± SD, *n* = 8.

Cells/μL	Phenotype	Pre-exercise	Post-exercise	30-min post-exercise	%Δ pre- to post-exercise	Effect of time
Plasma cells	CD38^bright^CD138^+^	2.6 ± 5.6	5.4 ± 12.6	2.9 ± 6.2	58 ± 41	*F*_(1.00,7.00)_ = 1.25, *p* = 0.30, ηp^2^ = 0.15
	CD38^bright^CD138^+^CD45^−/dim^CD19^−^ (with light-chain restriction)	2.1 ± 5.5	4.7 ± 12.3	2.3 ± 6.1	121 ± 95	*F*_(1.00,7.00)_ = 1.18, *p* = 0.31, ηp^2^ = 0.14
	CD38^bright^CD138^+^CD45^+^CD19^+^ (with polyclonal light chains)	0.33 ± 0.19	0.33 ± 0.19	0.33 ± 0.20	9 ± 41	*F*_(2,14)_ = 0.01, *p* = 0.99, ηp^2^ = 0.001
B-cells	CD19^+^	100 ± 80	121 ± 84^b^	100 ± 87^c^	32 ± 34	*F*_(2,14)_ = 11.60, *p* = 0.001, ηp^2^ = 0.62
	CD19^+^CD20^+^	99 ± 80	120 ± 84^b^	99 ± 87^c^	33 ± 35	*F*_(2,14)_ = 11.56, *p* = 0.001, ηp^2^ = 0.62
	CD19^+^CD20^+^CD27^−^CD38^+^	50 ± 52	57 ± 57^a^	47 ± 55^c^	28 ± 36	*F*_(2,14)_ = 9.96, *p* = 0.002, ηp^2^ = 0.59
	CD19^+^CD20^+^CD27^+^CD38^+^	33 ± 32	39 ± 33^a^	36 ± 34	30 ± 28	*F*_(2,14)_ = 3.94, *p* = 0.044, ηp^2^ = 0.36
	CD19^+^CD20^+^CD27^+^CD38^−^	9.1 ± 5.6	14 ± 8.4^a^	10 ± 6.6	52 ± 46	*F*_(2,14)_ = 7.10, *p* = 0.007, ηp^2^ = 0.50
	CD19^+^CD20^+^Ig-Kappa	60 ± 49	75 ± 53^b^	60 ± 54^d^	36 ± 36	*F*_(2,14)_ = 13.30, *p* < 0.001, ηp^2^ = 0.66
	CD19^+^CD20^+^Ig-lambda	38 ± 30	44 ± 30	38 ± 32	27 ± 35	*F*_(2,14)_ = 3.64, *p* = 0.053, ηp^2^ = 0.34

ANOVA, analysis of variance. Clonal plasma cells were phenotyped as CD38^bright^CD138^+^CD45^−/dim^CD19^−^ with light-chain restriction and polyclonal plasma cells were phenotyped as CD38^bright^CD138^+^CD45^+^CD19^+^ with polyclonal light chains. Transitional-like B-cells were phenotyped as CD19^+^CD20^+^CD27^−^CD38^+^, plasma blasts were phenotyped as CD19^+^CD20^+^CD27^+^CD38^+^, and memory B-cells were phenotyped as CD19^+^CD20^+^CD27^+^CD38^−^.

a indicates a significant difference from pre-exercise at *p* < 0.05.

b indicates a significant difference from pre-exercise at *p* < 0.01.

c indicates a significant difference from post-exercise at *p* < 0.05.

d indicates a significant difference from post-exercise at *p* < 0.01.

As expected, total CD19^+^ B-cells were significantly elevated following cycling exercise (+32 ± 34%; *p* = 0.002). Additionally, B-cell subsets with a transitional-like phenotype (CD19^+^CD20^+^CD27^−^CD38^+^), a memory phenotype (CD19^+^CD20^+^CD27^+^CD38^−^), and a plasma blast phenotype (CD19^+^CD20^+^CD27^+^CD38^+^) were all significantly elevated following cycling exercise (+≥28%; *p* < 0.05). The effects of exercise on plasma cells and B-cells in MREM participants are displayed in Supplementary Tables 1 and 2, respectively.

### 3.3 NK-cell mobilisation during cycling exercise

Table 5 displays the summary data and statistical results for NK-cells and NK-cell subsets. Total NK-cell frequency significantly increased by 302 ± 183%, with a preferential mobilisation of NK-cells capable of ADCC (CD3^−^CD56^+^CD16^+^, +348 ± 220%), mature CD3^−^CD56^+^CD57^+^ NK-cells (+339 ± 209%), and mature effector CD3^−^CD56^+^CD57^+^CD16^+^ NK-cells (+349 ± 236%) (*F*_(1.0,7.0)_ ≥ 10.68, *p* ≤ 0.013, ηp^2^ ≥ 0.60) following cycling exercise.

**Table 5:** Natural Killer (NK)-cell subsets pre-exercise, post-exercise, and 30-min post-exercise in participants with smouldering multiple myeloma with percentage change (%Δ) pre-to post-exercise and main effect of time from repeated measures ANOVA. Data are mean ± SD, *n* = 8.

CD3^−^CD56^+^ cells/μL	Pre-exercise	Post-exercise	30-min post-exercise	%Δ pre- to post-exercise	Effect of time
Total	53 ± 21	203 ± 119^a^	36 ± 14^a^,^c^	302 ± 183	*F*_(1.02,7.16)_ = 14.66, *p* = 0.006, ηp^2^ = 0.68
CD16^+^	46 ± 20	190 ± 117^a^	30 ± 12^a^,^c^	348 ± 220	*F*_(1.02,7.15)_ = 14.04, *p* = 0.007, ηp^2^ = 0.67
CD38^+^	50 ± 19	191 ± 116^a^	34 ± 14^a^,^c^	305 ± 199	*F*_(1.02,7.15)_ = 13.82, *p* = 0.007, ηp^2^ = 0.66
CD38^+^CD16^+^	43 ± 19	177 ± 113^a^	27 ± 11^a^,^c^	352 ± 239	*F*_(1.02,7.16)_ = 13.19, *p* = 0.008, ηp^2^ = 0.65
CD38^+^CD16^−^	7 ± 2	16 ± 8^b^	6 ± 3^d^	145 ± 94	*F*_(1.06,7.44)_ = 21.81, *p* = 0.002, ηp^2^ = 0.76
CD38^−^CD16^+^	2 ± 2	6 ± 7	2 ± 1	135 ± 113	*F*_(1.01,7.03)_ = 3.85, *p* = 0.090, ηp^2^ = 0.36
CD57^+^	31 ± 14	133 ± 92^a^	20 ± 11^a^,^c^	339 ± 209	*F*_(1.01,7.09)_ = 11.83, *p* = 0.010, ηp^2^ = 0.63
CD57^−^	22 ± 8	70 ± 32^a^	16 ± 4^d^	247 ± 145	*F*_(1.06,7.43)_ = 19.92, *p* = 0.002, ηp^2^ = 0.74
CD57^+^CD16^+^	30 ± 14	126 ± 92^a^	19 ± 10^a^,^c^	349 ± 236	*F*_(1.01,7.08)_ = 10.68, *p* = 0.013, ηp^2^ = 0.60
CD57^−^CD16^+^	17 ± 8	59 ± 32^a^	11 ± 3^a^,^c^	299 ± 200	*F*_(1.05,7.32)_ = 16.01, *p* = 0.005, ηp^2^ = 0.70
CD56^dim^	50 ± 20	194 ± 116^a^	33 ± 13^a^,^c^	313 ± 190	*F*_(1.02,7.15)_ = 14.60, *p* = 0.006, ηp^2^ = 0.68
CD56^dim^CD38^+^CD16^+^	42 ± 18	174 ± 110^a^	27 ± 11^a^,^c^	359 ± 245	*F*_(1.02,7.14)_ = 13.36, *p* = 0.008, ηp^2^ = 0.66
CD56^dim^CD38^+^CD16^−^	5 ± 2	13 ± 7^a^	4 ± 3^c^	170 ± 126	*F*_(1.05,7.33)_ = 15.85, *p* = 0.005, ηp^2^ = 0.69
CD56^dim^CD38^−^CD16^+^	3 ± 2	6 ± 7	2 ± 1	124 ± 122	*F*_(1.05,7.35)_ = 3.23, *p* = 0.113, ηp^2^ = 0.32
CD56^bright^	3 ± 2	8 ± 4^a^	3 ± 1^c^	145 ± 80	*F*_(1.09,7.65)_ = 12.97, *p* = 0.007, ηp^2^ = 0.65
CD56^bright^CD38^+^CD16^+^	1 ± 1	3 ± 3	1 ± 1	224 ± 182	*F*_(1.11,7.79)_ = 5.85, *p* = 0.040, ηp^2^ = 0.46
CD56^bright^CD38^+^CD16^−^	2 ± 1	3 ± 2^a^	2 ± 1^d^	115 ± 71	*F*_(1.13,7.92)_ = 17.14, *p* = 0.003, ηp^2^ = 0.71
CD56^bright^CD38^−^CD16^+^	0.1 ± 0.1	0.2 ± 0.3	0.1 ± 0.1	182 ± 232	*F*_(2,14)_ = 2.38, *p* = 0.15, ηp^2^ = 0.25

ANOVA, analysis of variance.

a indicates a significant difference from pre-exercise at *p* < 0.05.

b indicates a significant difference from pre-exercise at *p* < 0.01.

c indicates a significant difference from post-exercise at *p* < 0.05.

d indicates a significant difference from post-exercise at *p* < 0.01.

Given that NK-cells expressing CD38 may be susceptible to daratumumab-mediated NK-cell fratricide, we also explored the effects of cycling exercise on CD38^+^ NK-cells. In the SMM cohort, significant elevations were observed for CD3^−^CD56^+^CD38^+^ NK-cells (+305 ± 199%), CD3^−^CD56^+^CD38^+^CD16^−^ NK-cells (+145 ± 94%), and CD3^−^CD56^+^CD38^+^CD16^+^ NK-cells (+352 ± 239%) following cycling exercise (*F*_(1.0,7.0)_ ≥ 13.19, *p* ≤ 0.008, ηp^2^ ≥ 0.65). The effects of exercise on NK-cell subsets in MREM participants can be seen in Supplementary Table 3.

### 3.4 Monocyte mobilisation during cycling exercise

The summary data and statistical results for monocyte subsets are displayed in Table 6. There was a significant increase in the number of non-classical (HLA-DR^+^CD14^dim^CD16^+^) monocytes (+125 ± 89%), intermediate (HLA-DR^+^CD14^+^CD16^+^) monocytes (+48 ± 26%), classical (HLA-DR^+^CD14^+^CD16^dim^) monocytes (+41 ± 24%), and effector (HLA-DR^+^CD14^+^CD32^+^) monocytes (+41 ± 26%) following cycling exercise (*F*_(2,14)_ ≥ 11.98, *p* ≤ 0.006, ηp^2^ ≥ 0.63). The effects of exercise on MREM monocyte subsets are displayed in Supplementary Table 4.

**Table 6:** Monocyte and MDSC subsets pre-exercise, post-exercise, and 30-min post-exercise in participants with smouldering multiple myeloma, with percentage change (%Δ) pre-to post-exercise and main effect of time from repeated measures ANOVA. Data are mean ± SD, *n* = 8.

Cells/μL	Phenotype	Pre-exercise	Post-exercise	30-min post-exercise	%Δ Pre-Post	Main effect of time
Non-classical	HLA-DR ^+^ CD14^dim^CD16^+^	32 ± 17	63 ± 29^b^	31 ± 19^e^	125 ± 89	*F*_(2,14)_ = 21.73, *p* < 0.001, ηp^2^ = 0.76
Intermediate	HLA-DR^+^CD14^+^CD16^+^	32 ± 17	46 ± 20^b^	29 ± 14^d^	48 ± 26	*F*_(2,14)_ = 11.98, *p* < 0.001, ηp^2^ = 0.63
Classical	HLA-DR^+^CD14^+^CD16^−^	275 ± 52	388 ± 106^a^	271 ± 69^e^	41 ± 24	*F*_(2,14)_ = 17.00, *p* < 0.001, ηp^2^ = 0.71
Effector	HLA-DR^+^CD14^+^CD32^+^	322 ± 78	458 ± 153^a^	311 ± 88^d^	41 ± 26	*F*_(1.19,8.34)_ = 12.72, *p* = 0.006, ηp^2^ = 0.65
MDSCs	HLA-DR^−^CD33^+^	62 ± 22	90 ± 27^c^	62 ± 22^f^	49 ± 23	*F*_(1.07,7.47)_ = 46.80, *p* < 0.001, ηp^2^ = 0.87
M-MDSCs	HLA-DR^−^CD33^+^CD14^+^	21 ± 21	28 ± 36	19 ± 17	17 ± 63	*F*_(1.02,7.15)_ = 1.17, *p* = 0.32, ηp^2^ = 0.14
PMN-MDSCs	HLA-DR^−^CD33^+^CD14^−^	41 ± 27	58 ± 31	42 ± 25	43 ± 45	*F*_(1.03,7.18)_ = 6.12, *p* = 0.041, ηp^2^ = 0.47

ANOVA, analysis of variance; MDSCs, myeloid-derived suppressor cells; M-MDSCs, monocytic myeloid-derived suppressor cells; PMN-MDSCs, polymorphonuclear myeloid-derived suppressor cells.

a indicates a significant difference from pre-exercise at *p* < 0.05.

b indicates a significant difference from pre-exercise at *p* < 0.01.

c indicates a significant difference from pre-exercise at *p* < 0.001.

d indicates a significant difference from post-exercise at *p* < 0.05.

e indicates a significant difference from post-exercise at *p* < 0.01.

f indicates a significant difference from post-exercise at *p* < 0.001.

### 3.5 ADCC changes in response to cycling exercise

**Fig. 4:**
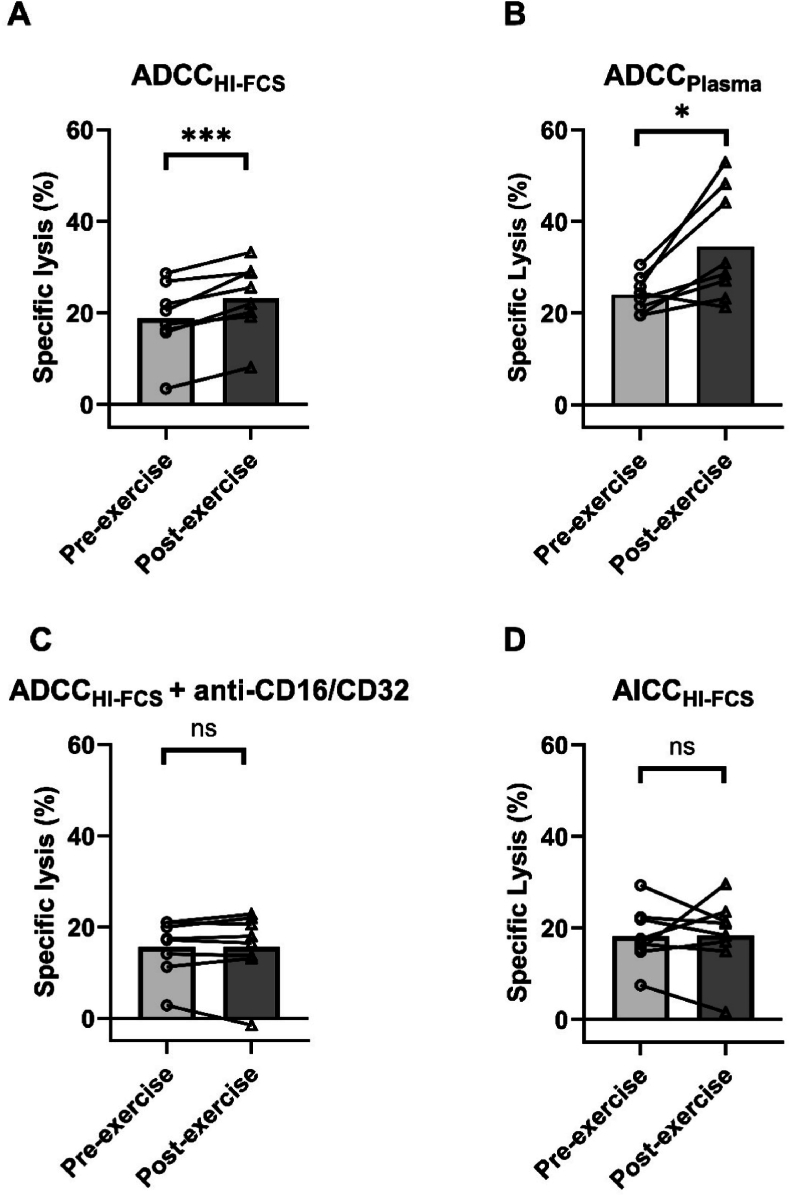
Specific lysis of RPMI-8226 cells in SMM participants pre-exercise (light grey bars) and post-exercise (dark grey bars). **A)** Specific lysis of RPMI-8226 cells cultured with PBMCs and HI-FCS mediated by daratumumab (ADCC_HI-FCS_). **B)** Specific lysis of RPMI-8226 cells cultured with PBMCs and time-point matched autologous plasma mediated by daratumumab (ADCC_Plasma_). **C)** Specific lysis of RPMI-8226 cells cultured with PBMCs and HI-FCS mediated by daratumumab and in the presence of CD16 and CD32 blocking antibodies (ADCC_HI-FCS_ + anti-CD16/CD32). **D)** Specific lysis of RPMI-8226 cells cultured with PBMCs and HI-FCS independent of daratumumab (AICC_HI-FCS_). ∗indicates a significant difference at *p* < 0.05, ∗∗∗indicates a significant difference at *p* < 0.001. ADCC, antibody-dependent cellular cytotoxicity; AICC, antibody-independent cellular cytotoxicity; HI-FCS, heat-inactivated foetal calf serum; ns, non-significant; SMM, smouldering multiple myeloma; PBMCs, peripheral blood mononuclear cells. Data are group means with individual responses overlaid, *n* = 8.

A significant increase was observed pre- to post-exercise in ADCC-mediated RPMI-8226 lysis when cultured with HI-FCS (ADCC_HI-FCS_) from 18.8% to 23.3%, respectively (*t*_(7)_ = 5.57, *p* < 0.001, *d* = 1.97) (Fig. 4A), and separately when cultured with time-point matched plasma (ADCC_Plasma_) increasing from 24.1% to 34.1% pre- to post-exercise, respectively (*t*_(7)_ = 2.82, *p* = 0.026, *d* = 1.00) (Fig. 4B), with no differences in ADCC + anti-CD16/32 (*t*_(7)_ = −0.07, *p* = 0.95, *d* = 0.02) (Fig. 4C), or AICC (*t*_(7)_ = 0.10, *p* = 0.92, *d* = 0.04) (Fig. 4D). Additionally, ADCC_Plasma_ post-exercise (34.1%) was significantly greater than ADCC_HI-FCS_ post-exercise (23.3%) (*t*_(7)_ = 3.72, *p* = 0.007, *d* = 1.31), with a trend towards a difference pre-exercise (*t*_(7)_ = 2.10, *p* = 0.07, *d* = 0.75).

In a series of other exploratory experiments, repeated measures ANOVA revealed no significant effects of time (pre-, post- and 30-min post-exercise) for whole blood ADCC (ADCC_WB_) (*F*_(1.18,8.25)_ = 1.81, *p* = 0.22, ηp^2^ = 0.21) or whole blood AICC (AICC_WB_) (*F*_(2,14)_ = 2.56, *p* = 0.11, ηp^2^ = 0.27) against RPMI-8226 cells (Supplementary Fig. 3).

### 3.6 T-cell mobilisation during cycling exercise

We also characterised the effects of a single bout of cycling 15% above anaerobic threshold on T-cell subsets, the summary data and statistical results of which can be seen in Table 7. As expected, following cycling exercise there were significant elevations in the frequency of total CD3^+^ T-cells (+50 ± 23%), CD3^+^CD8^+^ T-cells (+76 ± 40%), and CD3^+^CD4^+^ T-cells (+33 ± 20%) (*F*_(2,14)_ ≥ 10.24, *p* ≤ 0.012, ηp^2^ ≥ 0.59). There were no changes to the antigen expression – measured by median fluorescence intensity – of PD-1 or CTLA-4 on CD3^+^CD8^+^ T-cells (*F*_(2,14)_ = 1.54, *p* = 0.25, ηp^2^ = 0.18 and *F*_(1.06,7.40)_ = 1.33, *p* = 0.29, ηp^2^ = 0.16, respectively) or on CD3^+^CD4^+^ T-cells (*F*_(2,14)_ = 0.44, *p* = 0.65, ηp^2^ = 0.06 and *F*_(1.02,7.13)_ = 0.68, *p* = 0.44, ηp^2^ = 0.09, respectively). The effects of exercise on T-cell subsets in MREM participants can be seen in Supplementary Table 5.

**Table 7:** T-cell subsets pre-exercise, post-exercise, and 30-min post-exercise in participants with smouldering multiple myeloma with percentage change (%Δ) pre-to post-exercise and main effect of time from repeated measures ANOVA. Data are mean ± SD, *n* = 8.

CD3^+^ T-cells/μL	Pre-exercise	Post-exercise	30-min post-exercise	%Δ pre- to post-exercise	Effect of time
Total	1150 ± 483	1714 ± 740^b^	1113 ± 616^d^	50 ± 23	*F*_(2,14)_ = 19.22, *p* < 0.001, ηp^2^ = 0.76
CD8^+^	372 ± 243	654 ± 474^a^	354 ± 268^c^	76 ± 40	*F*_(1.09,7.65)_ = 10.24, *p* = 0.012, ηp^2^ = 0.59
CD8^+^CD28^+^CD57^−^	116 ± 63	155 ± 89^a^	125 ± 85	33 ± 28	*F*_(2,14)_ = 7.32, *p* = 0.007, ηp^2^ = 0.51
CD8^+^CD28^−^CD57^+^	155 ± 164	362 ± 373	126 ± 154	138 ± 40	*F*_(1.02,7.15)_ = 7.04, *p* = 0.032, ηp^2^ = 0.50
CD8^+^PD-1^+^	85 ± 48	138 ± 89^a^	87 ± 61	62 ± 38	*F*_(2,14)_ = 9.08, *p* = 0.003, ηp^2^ = 0.57
CD8^+^CTLA-4^+^	178 ± 111	314 ± 192^a^	155 ± 122^c^	84 ± 53	*F*_(1.10,7.70)_ = 16.29, *p* = 0.004, ηp^2^ = 0.70
CD4^+^	729 ± 318	972 ± 430^a^	717 ± 401^c^	33 ± 20	*F*_(2,14)_ = 12.92, *p* < 0.001, ηp^2^ = 0.65
CD4^+^CD28^+^CD57^−^	447 ± 226	638 ± 318	425 ± 285	59 ± 89	*F*_(2,14)_ = 5.75, *p* = 0.015, ηp^2^ = 0.45
CD4^+^CD28^−^CD57^+^	67 ± 138	54 ± 83	25 ± 34	75 ± 80	*F*_(2,14)_ = 0.61, *p* = 0.56, ηp^2^ = 0.08
CD4^+^PD-1^+^	170 ± 106	211 ± 124^a^	146 ± 115	30 ± 30	*F*_(2,14)_ = 4.10, *p* = 0.040, ηp^2^ = 0.37
CD4^+^CTLA-4^+^	242 ± 159	293 ± 227	188 ± 118	16 ± 33	*F*_(2,14)_ = 3.57, *p* = 0.056, ηp^2^ = 0.34

ANOVA, analysis of variance; PD-1, programmed cell death protein-1; CTLA-4, cytotoxic T-lymphocyte associated protein-4.

a indicates a significant difference from pre-exercise at *p* < 0.05.

b indicates a significant difference from pre-exercise at *p* < 0.01.

c indicates a significant difference from post-exercise at *p* < 0.05.

d indicates a significant difference from post-exercise at *p* < 0.01.

### 3.7 Myeloid-derived suppressor cell mobilisation during cycling exercise

As shown in Table 6, a significant effect of time was observed on total (HLA-DR^−^CD33^+^) MDSCs (*F*_(1.07,7.47)_ = 46.80, *p* < 0.001, ηp^2^ = 0.87), and polymorphonuclear (PMN)-MDSCs (HLA-DR^−^CD33^+^CD14^−^) (*F*_(1.03,7.18)_ = 6.12, *p* = 0.041, ηp^2^ = 0.47). Total MDSCs significantly increased pre- to post-exercise by 49 ± 23% (*p* < 0.001), with a trend observed for an increase in PMN-MDSCs pre- to post-exercise (+43 ± 45%; *p* = 0.076), with no effects observed on monocytic (M)-MDSCs (HLA-DR^−^CD33^+^CD14^+^) (*F*_(1.02,7.15)_ = 1.17, *p* = 0.32, ηp^2^ = 0.14) (Table 6). The effects of exercise on MREM MDSC subsets are presented in Supplementary Table 3.

## 4 Discussion

The primary aim of this study was to characterise the effects of an individual bout of cycling 15% above anaerobic threshold on the frequency of leukocytes in the blood of people with SMM and to determine whether immunomodulatory changes arising from exercise have the potential to alter the efficacy of daratumumab. As expected, total CD3^−^CD56^+^ NK-cells, effector CD3^−^CD56^+^CD16^+^ NK-cells, non-classical HLA-DR^+^CD14^dim^CD16^+^, and HLA-DR^+^CD14^+^CD32^+^ monocytes were elevated in blood immediately post-exercise. Additionally, our *in vitro* assay showed an increase in daratumumab-mediated ADCC activity when PBMCs isolated from participants were incubated with RPMI-8226 plasmacytoma cells, which was then nullified in the presence of antibodies blocking CD16 and CD32 receptors. However, there was no change in the frequency of circulating plasma cells. We also observed an elevated number of CD3^−^CD56^+^CD38^+^ NK-cells in blood, which may render these cells more susceptible (i.e., as an off-tumour target) to daratumumab itself.

To the best of our knowledge, this is the first study to assess whether an individual bout of cycling 15% above anaerobic threshold could be used as a method of mobilising clonal plasma cells. We tested this in SMM and detected clonal plasma cells – typified as CD38^bright^CD138^+^CD45^−/dim^CD19^−^ with light chain restriction – in 75% (6/8) of our participants with SMM pre-exercise, and in 100% (8/8) of SMM participants immediately following exercise (range: 0.115–15.660 clonal plasma cells/μL and 0.006–35.229 clonal plasma cells/μL, respectively). There were no changes in the numbers of total, or clonal plasma cells pre- to post-exercise. A strong effect was observed for total, and clonal plasma cell mobilisation, however any change pre- to immediately post-exercise could be considered modest (+3 cells/μL). In the absence of clonal plasma cell mobilisation into blood in response to exercise and given that the bulk of myeloma cells reside in the bone marrow (Kyle et al., 2003), it may be that a preferential redistribution of NK-cells and monocytes to bone after exercise – as is seen with T-cells (Krüger et al., 2008) – is a more viable means of enhancing daratumumab ADCC against myeloma plasma cells *in vivo*. Previous mouse models have demonstrated that immediately following an individual bout of vigorous running exercise, the frequency of T-cells is elevated in the bone marrow, thus indicating that immune effector cells may have the potential to migrate to bone marrow in response to exercise (Krüger et al., 2008). Moreover, a separate mouse model showed that 6-weeks of regular physical activity (i.e., frequent longer-term exercise training) increases the infiltration of NK-cells into the bone marrow (Pedersen et al., 2016). Therefore, a more viable means of enhancing daratumumab efficacy against myeloma cells may be to harness individual bouts of exercise to induce trafficking of effector immune cells (such as NK-cells and monocytes) to the bone marrow, where myeloma plasma cells reside, thus potentiating enhancement of daratumumab efficacy.

An important finding from this study is the observation that cycling exercise induced a profound mobilisation of NK-cells expressing CD38, which could be considered counterproductive for myeloma patients receiving daratumumab (Collier-Bain et al., 2023). During daratumumab therapy *in vivo*, NK-cell frequency decreases rapidly upon exposure to daratumumab, and remain at suboptimal levels throughout therapy, before beginning to recover ∼3-months following the completion of therapy (Casneuf et al., 2017). This may in part be due to NK-cell fratricide, whereby, daratumumab activates NK-cells to target neighbouring NK-cells expressing CD38 (Wang et al., 2018). Here we show that an individual bout of cycling 15% above anaerobic threshold induces a substantial increase in total CD3^−^CD56^+^CD38^+^ NK-cells (+305%), with a preferential increase to CD3^−^CD56^+^CD38^+^CD16^+^ NK-cells (+352%). The latter of which make up approximately 87% of the total NK-cells mobilised, and present as a double-edged sword, with the capacity to induce ADCC against myeloma cells, and being susceptible to ADCC by neighbouring NK-cells (i.e., fratricide). Therefore, it is important to consider that exercising patients with myeloma during daratumumab treatment could result in greater overall NK-cell reductions. However, this theory requires validation in future studies, including in patients receiving daratumumab. Notably, in the GEN501 and SIRIUS trials – where NK-cells were shown to rapidly decrease in number (Casneuf et al., 2017) – daratumumab as a single agent was still able to induce favourable overall response rates (36% and 29%, respectively) (Lokhorst et al., 2015; Lonial et al., 2016) and it was concluded that the NK-cells that remain are still capable of inducing ADCC (Casneuf et al., 2017) and may reflect the CD56^+^CD38^−^CD16^+^ NK-cell portion, which represent 3% of total NK-cells mobilised herein, and may increase by 135 ± 113% following cycling 15% above anaerobic threshold in SMM; and in *n* = 1 MREM participant who received daratumumab maintenance therapy we observed a 218% increase.

This study shows for the first time that an individual bout of cycling 15% above anaerobic threshold enhances the efficacy of daratumumab against RPMI-8226 cells *in vitro*. We showed that the addition of an anti-CD16 and anti-CD32 blocking antibody greatly diminished the effects of daratumumab, which, when coupled with no change observed for AICC, suggests that the increase in ADCC_HI-FCS_ was independent of a change in AICC and was dependent on the mobilisation of effector CD3^−^CD56^+^CD16^+^ NK-cells (+348%), non-classical monocytes (+125%), and HLA-DR^+^CD14^+^CD32^+^ monocytes (+41%). Indeed, we are unable to determine the different contributions of CD16^+^ and CD32^+^ effector cells on ADCC given that antibodies blocking CD16 and CD32 were simultaneously added in blocking experiments. However, it has been previously reported that blocking CD16 receptors alone induces greater ADCC inhibition compared to blocking CD32 receptors alone (Yeap et al., 2016), and HLA-DR^+^CD14^+^CD16^+^ effector cells were preferentially mobilised when compared to CD32^+^ effector cells herein. It is important to note here that MDSCs were also mobilised into blood immediately following cycling exercise, and these cells may inhibit ADCC through, for example, secretion of nitric oxide (Stiff et al., 2018). Separately, we also show herein that cycling 15% above anaerobic threshold increased circulating CD3^+^CD8^+^ T-cells (+76%). This is unsurprising given that previous research in healthy humans has shown increases of 24%, and 119% in CD3^+^CD8^+^ T-cells following low and vigorous intensity cycling, respectively (Campbell et al., 2009).

The preferential mobilisation of CD16^+^ NK-cells and monocytes is consistent with previous findings in CLL where a 254% increase in CD3^−^CD56^+^CD16^+^ NK-cells, and a 147% increase in non-classical monocytes was observed after a similar exercise protocol (Collier-Bain et al., 2024a). Additionally, in the aforementioned study, we showed that cycling 15% above anaerobic threshold increased the ADCC_HI-FCS_ activity of rituximab – an anti-CD20 antibody used to treat CLL (Hallek et al., 2010) – against autologous CLL cells with a change score of +129% pre- to post-exercise (Collier-Bain et al., 2024a). This change is approximately 92% greater than the change score to ADCC_HI-FCS_ herein (37%), however, our previous work utilised purified NK-cells and autologous CLL cells as targets in ADCC assays compared to the utilisation of total PBMCs and an immortal cell line as a target herein. Notably, although the percentage increase was greater in our previous work, the absolute percentage of specific lysis was less when compared to the present study pre-exercise (6% vs. 19%, respectively) and post-exercise (14% vs 23%, respectively). To explore a more physiologically relevant milieu, we also investigated whether ADCC_Plasma_ would elicit greater lysis of RPMI-8226 cells compared to ADCC_HI-FCS_. Post-exercise ADCC_Plasma_ was 58% greater than post-exercise ADCC_HI-FCS_, with a trend towards a difference pre-exercise (*p* = 0.07), which may represent a synergy between ADCC and other mechanisms-of-action of daratumumab, such as complement-dependent cytotoxicity (CDC) (de Weers et al., 2011). However, complement proteins – particularly C1q, which initiates CDC – were not measured in this study but do not appear to change following 45-min of running 15% above anaerobic threshold in healthy humans (Collier-Bain et al., 2024b). Adding to this, we explored the effects of cycling exercise on ADCC using whole blood as the source of effector cells, with no differences in ADCC_WB_ pre-, post-, or 30-min post-exercise. However, it is important to note that the assay used in our study was designed for PBMCs, and the investigation of whole blood was exploratory. As such, untouched whole blood was added in 100 μL volumes making up 50% of a 200 μL well. Thus, the concentration of immune cells in whole blood conditions was 50% of the concentration observed in whole blood *in vivo* and therefore fewer effector cells might be responsible for this result. On the other hand, isolated PBMCs were added in 100 μL volumes, but at 2 × the concentration of cells in the circulation. Thus, when added to a 200 μL well, the concentration of PBMCs within 200 μL was equivalent (i.e., 100%) to that observed in the circulation *in vivo* and therefore twice as much as the whole blood conditions in our assay.

A limitation to our study may be the heterogeneity in our sample, whereby participants had a variable number of circulating clonal plasma cells, which in part explains a lack of a significant finding in clonal plasma cell mobilisation immediately following cycling exercise. However, this heterogeneity in the number of clonal plasma cells herein is consistent with typical SMM populations, with previous research reporting ranges of circulating clonal plasma cells in SMM of 0.003–20,958 cells/μL (*n* = 109) (Termini et al., 2022) and 0.005–12.9 cells/μL (*n* = 25) (Sanoja-Flores et al., 2018). Notably, these previous studies employed the EuroFlow next generation flow cytometry approach which has greater sensitivity in detecting circulating clonal plasma cells compared to conventional flow cytometry (Flores-Montero et al., 2017), as used herein. Previous research using this approach, compared the frequency of circulating clonal plasma cells with paired bone marrow MRD in myeloma patients following treatment (Sanoja-Flores et al., 2019). The aforementioned study showed that every patient with clonal plasma cells in blood, was also MRD^+^ in bone marrow, with 40% of MRD^+^ cases showing undetectable circulating clonal plasma cells (Sanoja-Flores et al., 2019). Thus, the strong effect size observed herein may still have important clinical implications for patients in myeloma remission. Specifically, an individual bout of cycling 15% above anaerobic threshold may move a portion of MRD cells from the bone marrow into the blood, rendering them detectable by EuroFlow flow cytometry. Indeed, we are unable to confirm this as only *n* = 4 remission patients were included in this study as an exploratory sub-analysis. However, we focussed on SMM to provide preliminary evidence of plasma cell mobilisation given that previous research reports that the majority of SMM patients have detectable circulating plasma cells (Sanoja-Flores et al., 2018; Termini et al., 2022). It would be beneficial for future research to apply the EuroFlow next generation flow cytometry approach in an exercise setting to further our understanding of plasma cell mobilisation, specifically for the non-invasive detection of MRD. Another limitation to this study, and particularly, the plasma cell findings, is that the intensity of exercise may have been insufficient to stimulate a large magnitude of change in plasma cell frequency. Considering that exercise-induced leukocytosis is intensity-dependent, it is possible that the increases in immune cell subsets in our study may be more pronounced if an exercise protocol with a greater intensity was used. Additionally, NK-cell phenotypic investigations were conducted on thawed PBMCs. We used cryopreserved cells, rather than fresh whole blood, because we sought to decide on an NK-cell panel after determining the results of plasma cell mobilisation and ADCC assay results. Lastly, it is important to acknowledge that this was an exploratory pilot study and therefore, another limitation to the study was the small sample size. Although we were able to demonstrate a significant lymphocytosis and subsequent improvement in daratumumab-mediated ADCC activity, the lack of a significant finding in plasma cell mobilisation during the exercise bout could be attributed, in part, to the small sample size. Nevertheless, as noted earlier, it appears that any exercise-induced mobilisation of plasma cells is modest in SMM (+3 cells/μL).

Despite the limitations of the study, the results have other important clinical implications. For example, we show that cycling 15% above anaerobic threshold mobilises CD3^−^CD56^+^CD16^+^ NK-cells and non-classical monocytes, improving daratumumab-mediated ADCC by a change score of nearly 40% *in vitro*. It may be the case that a portion of these immune cells travel to bone marrow during and/or following exercise (Pedersen et al., 2016), increasing the ratio of NK-cells and monocytes to myeloma cells, previously shown to induce greater daratumumab-mediated ADCC (Nijhof et al., 2015). However, this needs to be validated *in vivo* given that we also show that cycling 15% above anaerobic threshold profoundly increases CD3^−^CD56^+^CD38^+^ NK-cells in blood, and these cells may be susceptible to daratumumab-mediated ADCC via fratricide – a potentially deleterious off-tumour effect. Importantly the increase in CD3^−^CD56^+^CD38^+^ cells was greater than the change in clonal plasma cells both in terms of absolute change (+141 cells/μL vs +3 cells/μL, respectively) and percentage change (+305% vs +121%, respectively). Thus, *in vivo*, it might be the case that exercise during daratumumab infusions may exacerbate the reduction in absolute NK-cell frequency (via daratumumab mediated NK-cell fratricide) and may outweigh the benefits arising from any modest clonal plasma cell mobilisation, highlighting the importance of exercise timing during therapy, as described recently (Collier-Bain et al., 2023). This theory requires validation, especially given that daratumumab's efficacy is still favourable despite the reductions to NK-cells *in vivo*.

In summary, our findings show that an individual bout of cycling 15% above anaerobic threshold transiently increased the frequency of CD3^−^CD56^+^CD16^+^ NK-cells, HLA-DR^+^CD14^dim^CD16^+^ monocytes, and HLA-DR^+^CD14^+^CD32^+^ monocytes in the blood of people with SMM, resulting in enhanced efficacy of daratumumab-mediated ADCC *in vitro*. However, no changes were observed for total, and clonal plasma cells. Notably, we observed an increase in the frequency of CD3^−^CD56^+^CD38^+^ NK-cells, which might render NK-cells more susceptible to daratumumab-mediated fratricide – a potentially deleterious off-tumour effect of daratumumab. Future research should consider the impact of exercise – and its timing relative to daratumumab therapy – on immune cells and clonal plasma cells *in vivo*. For example, it is important to determine whether there is a preferential killing of CD38^+^ NK-cells over myeloma cells during bouts of exercise, and to assess this impact of exercise in a larger, more diverse cohort of patients before recommendations of exercise timing during therapy can be made.

## Funding

This study was supported by 10.13039/501100000289Cancer Research UK (Grant number: C60293/A28497).

## CRediT authorship contribution statement

**Harrison D. Collier-Bain:** Writing – review & editing, Writing – original draft, Project administration, Methodology, Investigation, Formal analysis, Data curation. **Annabelle Emery:** Methodology, Investigation. **Frankie F. Brown:** Methodology, Investigation. **Adam J. Causer:** Methodology. **Rebecca Oliver:** Project administration, Investigation. **Rachel Eddy:** Investigation. **Shoji Leach:** Investigation. **John Graby:** Investigation. **Daniel Augustine:** Investigation. **Sally Moore:** Supervision, Project administration, Methodology, Investigation, Funding acquisition. **Josephine Crowe:** Supervision, Project administration, Methodology, Investigation, Funding acquisition. **James Murray:** Supervision, Project administration, Methodology, Investigation. **James E. Turner:** Writing – review & editing, Supervision, Project administration, Methodology, Investigation, Funding acquisition. **John P. Campbell:** Writing – review & editing, Supervision, Project administration, Methodology, Investigation, Funding acquisition, Formal analysis, Conceptualization.

## Declaration of competing interest

The authors declare that the research was conducted in the absence of any commercial or financial relationships that could be construed as a potential conflict of interest.

## Data Availability

Data will be made available on request.
