# Supplementary material for: Characterising how a single bout of exercise in people with myeloma affects clonal plasma cell and immune effector cell frequency in blood, and daratumumab efficacy in vitro
Source: Brain Behav Immun Health. 2024 Sep 19;42:100865. doi: 10.1016/j.bbih.2024.100865 (PMC11472639; doi:10.1016/j.bbih.2024.100865)
Supplement: Multimedia component 1 [file mmc1.docx]

**Supplementary materials**

**Characterising how a single bout of exercise in people with myeloma affects clonal plasma cell and immune effector cell frequency in blood, and daratumumab efficacy *in vitro*.**

Harrison D. Collier-Bain^a^, Annabelle Emery^a^, Frankie F. Brown^a,b^, Adam J. Causer^a^, Rebecca Oliver^a,c^, Rachel Eddy^a^, Shoji Leach^a^, John Graby^a,d^, Daniel Augustine^c^, Sally Moore^c^, Josephine Crowe^c^, James Murray^c^, James E. Turner^a,d^, and John P. Campbell^a,f*^.

^a^ Department for Health, University of Bath, UK.

^b^ School of Applied Sciences, Edinburgh Napier University, Edinburgh, United Kingdom.

^c^ Department of Haematology, Royal United Hospitals Bath NHS Foundation Trust, UK.

^d^ Department of Cardiology, Royal United Hospitals Bath NHS Foundation Trust, UK.

^e^ School of Sport, Exercise and Rehabilitation Sciences, University of Birmingham, Birmingham, United Kingdom.

^f^ School of Medical and Health Sciences, Edith Cowan University, Perth, Australia.

* Correspondence to Dr. J. P. Campbell, Department for Health, University of Bath, UK, BA2 7AY; Tel: +44 (0)1225 385495; Email: J.Campbell@bath.ac.uk

**SUPPLEMENTARY FIGURES**


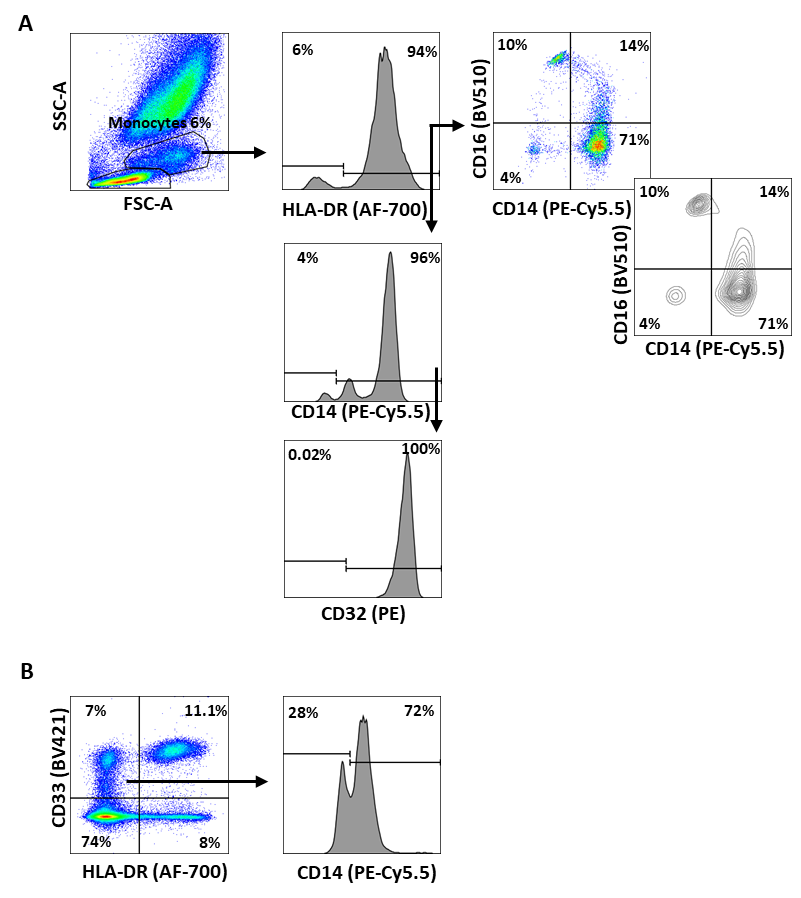


**Supplementary Figure 1.** Representative gating strategy for monocytes and myeloid-derived suppressor cells (MDSCs). **A)** Following the exclusion of doublets and debris (not shown), monocytes were identified in a SSC-A × FSC-A plot and distinguished from MDSCs by the expression of MHC class II receptor - HLA-DR using a histogram. Within the HLA-DR^+^ monocyte population, CD16 was plotted against CD14 to identify monocyte subsets including: non-classical (HLA-DR^+^CD14^dim^CD16^+^), intermediate (HLA-DR^+^CD14^+^CD16^+^), and classical (HLA-DR^+^CD14^+^CD16^−^) monocytes. Within the HLA-DR^+^ monocyte population, all CD14^+^ monocytes were identified using a histogram, followed by identification of HLA-DR^+^CD14^+^ monocytes expressing the effector receptor CD32. **B)** Lymphocytes and monocytes were combined using a Boolean ‘make OR gate’ function, and MDSCs were identified as HLA-DR^−^CD33^+^. Within the HLA-DR^−^CD33^+^ population, polymorphonuclear MDSCs were identified as CD14^−^ and monocytic MDSCs were identified as CD14^+^. SSC-A, side scatter-area; FSC-A forward scatter-area; HLA, human leukocyte antigen.


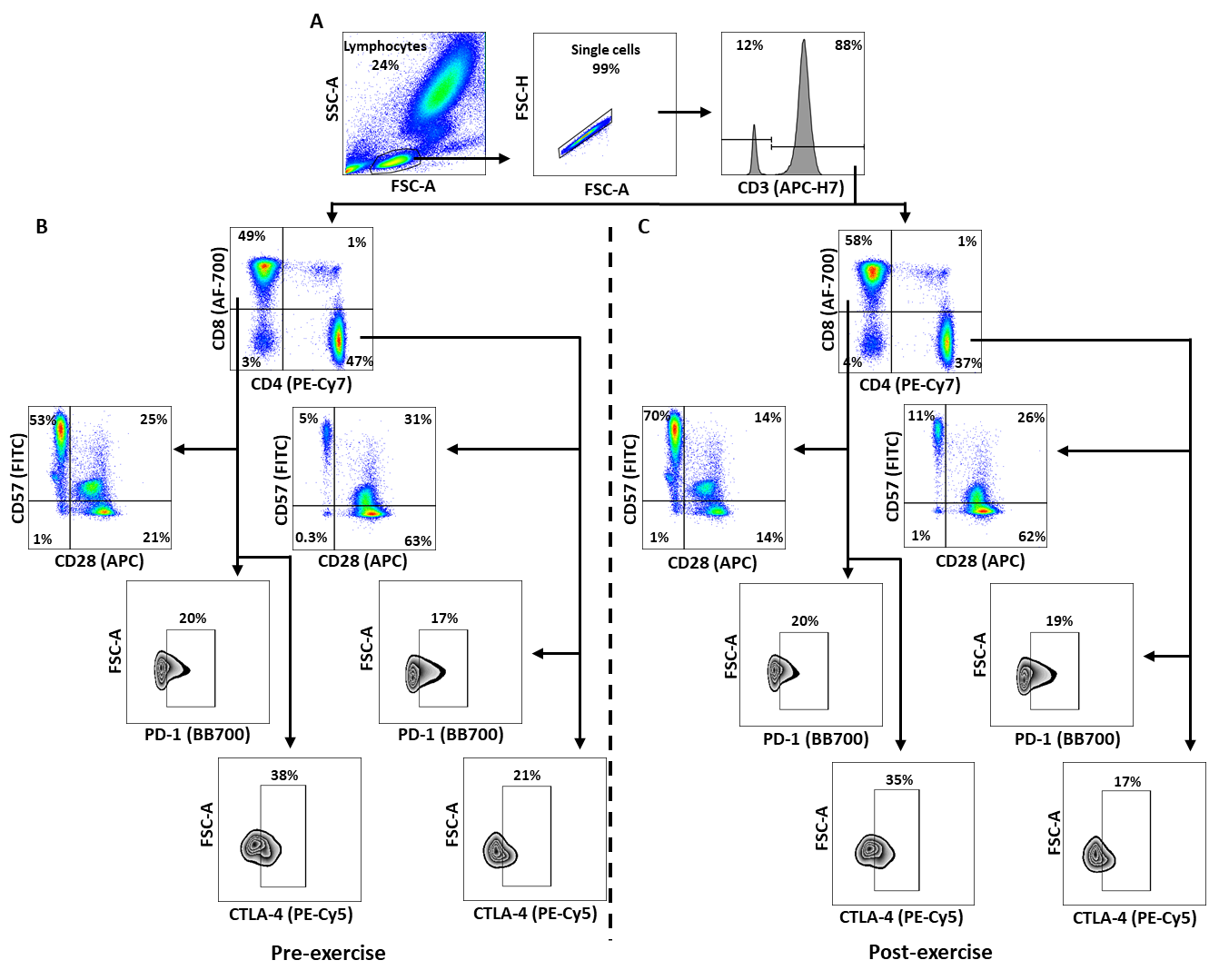
**Supplementary Figure 2.** Representative gating strategy for T-cells in SMM. **A)** Lymphocytes were identified in a SSC-A × FSC-A plot prior to exclusion of doublets in a FSC-H × FSC-A plot. Next, CD3^+^ T-cells were identified using a histogram. **B)** In a pre-exercise sample, a CD8 × CD4 plot was used to identify T-cell subsets. CD8^+^ and CD4^+^ T-cells were further gated to identify: early activated (CD28^+^CD57^−^), and senescent like (CD28^−^CD57^+^); PD-1^+^ T-cells; and CTLA-4^+^ T-cells. **C)** Represents the same gating strategy as ‘**B)**’ but in a post-exercise sample. SSC-A, side scatter-area; FSC-A, forward scatter-area; FSC-H, forward scatter-height; PD-1, programmed death cell-1; CTLA-4, cytotoxic T-lymphocyte-associated protein-4; SMM, smouldering multiple myeloma.


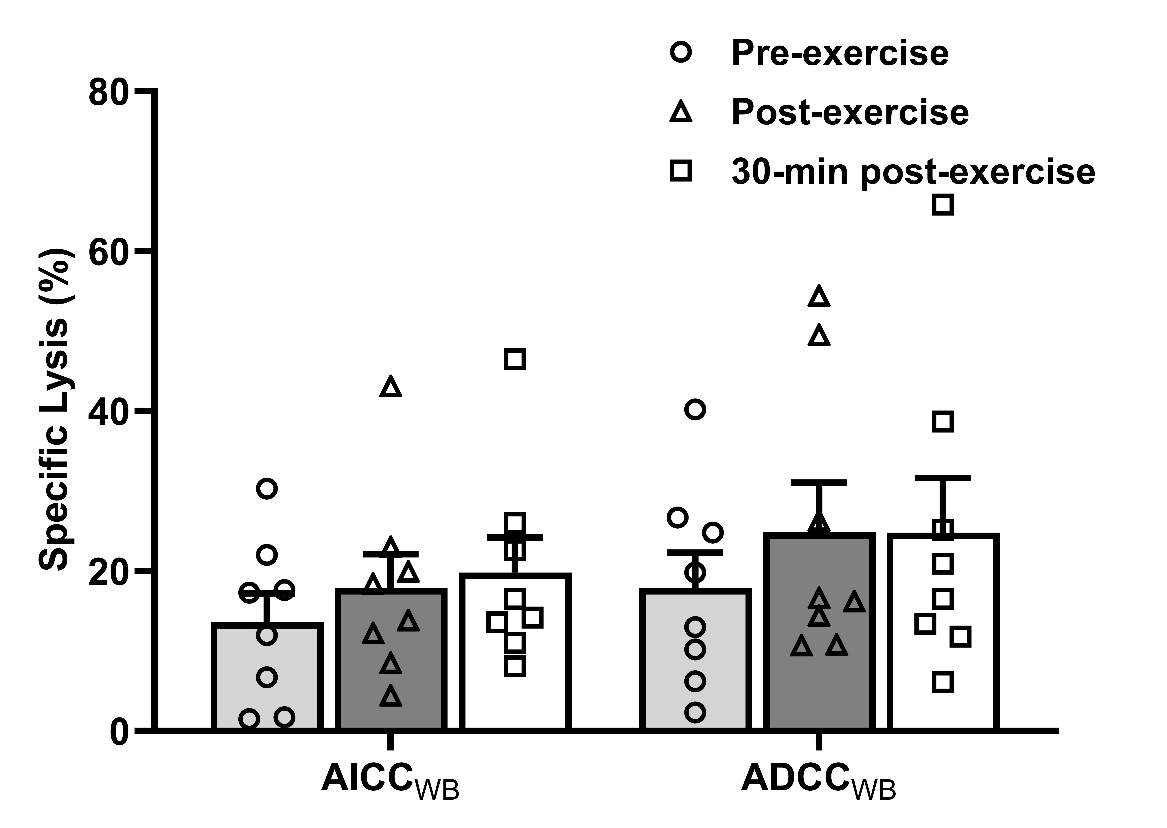


**Supplementary Figure 3.** Specific lysis of RPMI-8226 cells when cultured with whole blood (WB) independent of daratumumab (AICC_WB_), and mediated by daratumumab (ADCC_WB_) in SMM participants pre-exercise (light grey bars, open circles), post-exercise (dark grey bars, open triangles), and 30-min post-exercise (white bars, open squares). Data are mean ± SEM with individual responses overlaid, n = 8. AICC, antibody-independent cellular cytotoxicity; ADCC, antibody-dependent cellular cytotoxicity.

**SUPPLEMENTARY TABLES**

| **Supplementary Table 1.** Plasma cells pre-exercise, post-exercise, and 30-min post-exercise in participants in myeloma remission treated with lenalidomide (*n* = 3), or daratumumab (*n* = 1) maintenance therapy with percentage change (%Δ) pre- to post-exercise and main effect of time from repeated measures ANOVA for lenalidomide treated patients. Data are mean ± SD. | | | | | | |
| --- | --- | --- | --- | --- | --- | --- |
| Plasma cells/µL | Maintenance therapy | Pre-exercise | Post-exercise | 30-min post-exercise | %Δ pre- to post-exercise | Effect of time |
| Total | Lenalidomide | 0.81 ± 0.60 | 1.09 ± 0.44 | 0.68 ± 0.40^†^ | 56 ± 44 | *F*_(2,4)_ = 10.78, *p* = 0.024, ηp^2^ = 0.84 |
|  | Daratumumab | 0.005 | 0.021 | 0.016 | 294 |  |
| Clonal plasma cells | Lenalidomide | 0.000 ± 0.000 | 0.002 ± 0.004 | 0.002 ± 0.004 |  | *F*_(2,4)_ = 0.40, *p* = 0.69, ηp^2^ = 0.17 |
|  | Daratumumab | 0.000 | 0.000 | 0.000 |  |  |
| Polyclonal plasma cells | Lenalidomide | 0.73 ± 0.58 | 0.91 ± 0.44 | 0.61 ± 0.41^†^ | 44 ± 37 | *F*_(2,4)_ = 8.31, *p* = 0.038, ηp^2^ = 0.81 |
|  | Daratumumab | 0.005 | 0.005 | 0.010 | −2 |  |

**^†^**indicates a significant difference from post-exercise at *p* < 0.05 following *post hoc* Bonferroni comparisons. Plasma cells were phenotyped as CD38^bright^CD138^+^. Clonal plasma cells were phenotyped as CD38^bright^CD138^+^CD45^−/dim^CD19^−^ with light-chain restriction. Polyclonal plasma cells were phenotyped as CD38^bright^CD138^+^CD45^+^CD19^+^ with polyclonal light chains. ANOVA, analysis of variance.

| **Supplementary Table 2.** B-cell subsets pre-exercise, post-exercise, and 30-min post-exercise in participants in myeloma remission treated with lenalidomide (*n* = 3), or daratumumab (*n* = 1) maintenance therapy with percentage change (%Δ) pre- to post-exercise and main effect of time from repeated measures ANOVA for lenalidomide treated patients. Data are mean ± SD. | | | | | | |
| --- | --- | --- | --- | --- | --- | --- |
| B-cells/µL | Maintenance therapy | Pre-exercise | Post-exercise | 30-min post-exercise | %Δ pre- to post-exercise | Effect of time |
| CD19^+^ | Lenalidomide | 72 ± 45 | 91 ± 54 | 72 ± 41 | 28 ± 23 | *F*_(2,4)_ = 3.52, *p* = 0.13, ηp^2^ = 0.64 |
|  | Daratumumab | 7 | 10 | 7 | 55 |  |
| CD19^+^CD20^+^ | Lenalidomide | 69 ± 44 | 87 ± 54 | 69 ± 41 | 25 ± 25 | *F*_(2,4)_ = 2.86, *p* = 0.17, ηp^2^ = 0.59 |
|  | Daratumumab | 7 | 10 | 6 | 49 |  |
| CD19^+^CD20^+^CD27^−^CD38^+^ | Lenalidomide | 30 ± 24 | 36 ± 29 | 30 ± 25 | 23 ± 15 | *F*_(2,4)_ = 2.28, *p* = 0.22, ηp^2^ = 0.53 |
|  | Daratumumab | 0.006 | 0.018 | 0.000 | 176 |  |
| CD19^+^CD20^+^CD27^+^CD38^+^ | Lenalidomide | 30 ± 29 | 38 ± 33 | 28 ± 23 | 35 ± 24 | *F*_(2,4)_ = 2.28, *p* = 0.22, ηp^2^ = 0.53 |
|  | Daratumumab | 0.13 | 0.20 | 0.12 | 57 |  |
| CD19^+^CD20^+^CD27^+^CD38^−^ | Lenalidomide | 5 ± 3 | 7 ± 3 | 6 ± 3 | 66 ± 62 | *F*_(2,4)_ = 4.49, *p* = 0.10, ηp^2^ = 0.69 |
|  | Daratumumab | 4 | 6 | 4 | 50 |  |
| CD19^+^CD20^+^Ig-Kappa | Lenalidomide | 44 ± 29 | 55 ± 34 | 44 ± 26 | 25 ± 24 | *F*_(2,4)_ = 3.24, *p* = 0.15, ηp^2^ = 0.62 |
|  | Daratumumab | 4 | 5 | 3 | 47 |  |
| CD19^+^CD20^+^Ig-lambda | Lenalidomide | 25 ± 16 | 31 ± 21 | 25 ± 15 | 24 ± 26 | *F*_(2,4)_ = 2.24, *p* = 0.22, ηp^2^ = 0.53 |
|  | Daratumumab | 3 | 4 | 3 | 48 |  |

Transitional-like B-cells were phenotyped as CD19^+^CD20^+^CD27^−^CD38^+^, plasma blasts were phenotyped as CD19^+^CD20^+^CD27^+^CD38^+^, and memory B-cells were phenotyped as CD19^+^CD20^+^CD27^+^CD38^−^. ANOVA, analysis of variance.

| **Supplementary Table 3.** Natural Killer (NK)-cell subsets pre-exercise, post-exercise, and 30-min post-exercise in participants in myeloma remission with percentage change (%Δ) pre- to post-exercise and main effect of time from repeated measures ANOVA. Data are mean ± SD, *n* = 4. | | | | | |
| --- | --- | --- | --- | --- | --- |
| CD3-CD56^+^ cells/µL | Pre-exercise | Post-exercise | 30-min post-exercise | %Δ pre- to post-exercise | Effect of time |
| Total | 59 ± 38 | 185 ± 128 | 47 ± 35 | 197 ± 66 | *F*_(2,6)_ = 7.69, *p* = 0.022, ηp^2^ = 0.72 |
| CD16^+^ | 52 ± 34 | 163 ± 117 | 39 ± 30 | 202 ± 80 | *F*_(2,6)_ = 7.20, *p* = 0.025, ηp^2^ = 0.71 |
| CD38^+^ | 52 ± 35 | 172 ± 126 | 43 ± 34 | 203 ± 73 | *F*_(2,6)_ = 7.01, *p* = 0.027, ηp^2^ = 0.70 |
| CD38^+^CD16^+^ | 47 ± 32 | 151 ± 114 | 35 ± 29 | 190 ± 101 | *F*_(2,6)_ = 6.46, *p* = 0.032, ηp^2^ = 0.68 |
| CD38^+^CD16^−^ | 7 ± 5 | 20 ± 13 | 8 ± 6 | 186 ± 76 | *F*_(2,6)_ = 8.74, *p* = 0.017, ηp^2^ = 0.75 |
| CD38^−^CD16^+^ | 5 ± 3 | 12 ± 6 | 4 ± 2 | 157 ± 44 | *F*_(1.01,3.03)_ = 16.34, *p* = 0.004, ηp^2^ = 0.85 |
| CD57^+^ | 27 ± 20 | 98 ± 78 | 20 ± 17 | 239 ± 98 | *F*_(2,6)_ = 5.89, *p* = 0.038, ηp^2^ = 0.66 |
| CD57^−^ | 32 ± 19 | 87 ± 53 | 27 ± 18 | 165 ± 35 | *F*_(2,6)_ = 10.13, *p* = 0.012, ηp^2^ = 0.77 |
| CD57^+^CD16^+^ | 26 ± 19 | 93 ± 75 | 19 ± 17 | 237 ± 102 | *F*_(2,6)_ = 5.69, *p* = 0.041, ηp^2^ = 0.66 |
| CD57^−^CD16^+^ | 26 ± 15 | 71 ± 44 | 19 ± 13 | 172 ± 54 | *F*_(2,6)_ = 9.63, *p* = 0.013, ηp^2^ = 0.76 |
| CD56^dim^ | 54 ± 35 | 176 ± 123 | 43 ± 32 | 252 ± 80 | *F*_(2,6)_ = 7.69, *p* = 0.022, ηp^2^ = 0.72 |
| CD56^dim^CD38^+^CD16^+^ | 44 ± 30 | 148 ± 112 | 34 ± 30 | 206 ± 95 | *F*_(2,6)_ = 6.47, *p* = 0.032, ηp^2^ = 0.68 |
| CD56^dim^CD38^+^CD16^−^ | 4 ± 3 | 17 ± 11 | 7 ± 6 | 270 ± 137 | *F*_(2,6)_ = 8.41, *p* = 0.018, ηp^2^ = 0.74 |
| CD56^dim^CD38^−^CD16^+^ | 5 ± 3 | 11 ± 6 | 4 ± 2 | 159 ± 31 | *F*_(2,6)_ = 12.76, *p* = 0.007, ηp^2^ = 0.81 |
| CD56^bright^ | 5 ± 3 | 9 ± 6 | 4 ± 3 | 75 ± 43 | *F*_(2,6)_ = 6.33, *p* = 0.033, ηp^2^ = 0.68 |
| CD56^bright^CD38^+^CD16^+^ | 2 ± 1 | 4 ± 3 | 1 ± 1 | 78 ± 55 | *F*_(2,6)_ = 4.38, *p* = 0.067, ηp^2^ = 0.59 |
| CD56^bright^CD38^+^CD16^−^ | 3 ± 2 | 4 ± 3 | 2 ± 2 | 66 ± 25 | *F*_(2,6)_ = 5.36,l *p* = 0.046, ηp^2^ = 0.64 |
| CD56^bright^CD38^−^CD16^+^ | 0.1 ± 0.03 | 0.2 ± 0.1 | 0.1 ± 0.03 | 168 ± 212 | *F*_(2,6)_ = 2.58, *p* = 0.16, ηp^2^ = 0.46 |

ANOVA, analysis of variance.

| **Supplementary Table 4.** Monocyte subsets pre-exercise, post-exercise, and 30min post-exercise in participants in myeloma remission with percentage change (%Δ) pre- to post-exercise and main effect of time from repeated measures ANOVA. Data are mean ± SD, *n* = 4. | | | | | | |
| --- | --- | --- | --- | --- | --- | --- |
| Cells/µL | Phenotype | Pre-exercise | Post-exercise | 30min post-exercise | %Δ Pre-Post | Main effect of time |
| Non-classical | HLA-DR^+^CD14^dim^CD16^+^ | 12 ± 9 | 27 ± 30 | 12 ± 10 | 117 ± 89 | *F*_(2,6)_ = 2.11, *p* = 0.20, ηp^2^ = 0.41 |
| Intermediate | HLA-DR^+^CD14^+^CD16^+^ | 40 ± 30 | 66 ± 32 | 40 ± 24 | 97 ± 58 | *F*_(2,6)_ = 8.77, *p* = 0.017, ηp^2^ = 0.75 |
| Classical | HLA-DR^+^CD14^+^CD16^−^ | 313 ± 169 | 509 ± 216 | 304 ± 157 | 73 ± 45 | *F*_(2,6)_ = 12.16, *p* = 0.008, ηp^2^ = 0.80 |
| Effector | HLA-DR^+^CD14^+^CD32^+^ | 367 ± 203 | 607 ± 266 | 359 ± 184 | 76 ± 46 | *F*_(2,6)_ = 10.55, *p* = 0.011, ηp^2^ = 0.78 |
| MDSCs | HLA-DR^−^CD33^+^ | 50 ± 20 | 88 ± 40 | 59 ± 30 | 79 ± 41 | *F*_(1.01,3.03)_ = 9.07, *p* = 0.056, ηp^2^ = 0.75 |
| M-MDSCs | HLA-DR^−^CD33^+^CD14^+^ | 10 ± 11 | 17 ± 21 | 31 ± 39 | 57 ± 32 | *F*_(2,6)_ = 0.98, *p* = 0.43, ηp^2^ = 0.25 |
| PMN-MDSCs | HLA-DR^−^CD33^+^CD14^−^ | 40 ± 15 | 71 ± 35 | 32 ± 16 | 79 ± 47 | *F*_(1.00,3.00)_ = 4.36, *p* = 0.13, ηp^2^ = 0.59 |

ANOVA, analysis of variance; MDSCs, myeloid-derived suppressor cells; M-MDSCs, monocytic myeloid-derived suppressor cells; PMN-MDSCs, polymorphonuclear myeloid-derived suppressor cells; HLA, human leukocyte antigen.

| **Supplementary Table 5.** T-cell subsets pre-exercise, post-exercise, and 30-min post-exercise in myeloma remission with percentage change (%Δ) pre- to post-exercise and main effect of time from repeated measures ANOVA. Data are mean ± SD, *n* = 4. | | | | | |
| --- | --- | --- | --- | --- | --- |
| CD3^+^ T-cells/µL | Pre-exercise | Post-exercise | 30-min post-exercise | %Δ pre- to post-exercise | Effect of time |
| Total | 499 ± 122 | 868 ± 335 | 559 ± 177 | 71 ± 36 | *F*_(2,6)_ = 9.32, *p* = 0.014, ηp^2^ = 0.76 |
| CD8^+^ | 218 ± 114 | 436 ± 176 | 234 ± 88 | 110 ± 64 | *F*_(2,6)_ = 14.46, *p* = 0.005, ηp^2^ = 0.83 |
| CD8^+^CD28^+^CD57^−^ | 105 ± 23 | 172 ± 44 | 119 ± 28^†^ | 66 ± 38 | *F*_(2,6)_ = 12.91, *p* = 0.007, ηp^2^ = 0.81 |
| CD8^+^CD28^−^CD57^+^ | 65 ± 56 | 166 ± 110 | 65 ± 54 | 198 ± 110 | *F*_(2,6)_ = 9.17, *p* = 0.015, ηp^2^ = 0.75 |
| CD8^+^PD-1^+^ | 56 ± 45 | 106 ± 69 | 60 ± 35 | 100 ± 55 | *F*_(2,6)_ = 8.09, *p* = 0.020, ηp^2^ = 0.73 |
| CD8^+^CTLA-4^+^ | 62 ± 18 | 163 ± 93 | 66 ± 33 | 154 ± 79 | *F*_(1.00,3.01)_ = 7.51, *p* = 0.071, ηp^2^ = 0.72 |
| CD4^+^ | 245 ± 97 | 364 ± 244 | 291 ± 163 | 39 ± 38 | *F*_(1.02,3.06)_ = 2.46, *p* = 0.17, ηp^2^ = 0.45 |
| CD4^+^CD28^+^CD57^−^ | 231 ± 96 | 338 ± 226 | 276 ± 158 | 37 ± 36 | *F*_(1.02,3.07)_ = 2.47, *p* = 0.17, ηp^2^ = 0.45 |
| CD4^+^CD28^−^CD57^+^ | 1.4 ± 1.9 | 5.0 ± 8.5 | 2.0 ± 3.2 | 113 ± 155 | *F*_(1.01,3.03)_ = 1.04, *p* = 0.38, ηp^2^ = 0.26 |
| CD4^+^PD-1^+^ | 54 ± 24 | 83 ± 54 | 64 ± 37 | 43 ± 34 | *F*_(2,6)_ = 3.81, *p* = 0.086, ηp^2^ = 0.56 |
| CD4^+^CTLA-4^+^ | 11 ± 0.5 | 14 ± 5 | 11 ± 3 | 27 ± 44 | *F*_(2,6)_ = 1.87, *p* = 0.23, ηp^2^ = 0.38 |

^†^indicates a significant difference from post-exercise at *p* < 0.05 following *post hoc* Bonferroni comparisons. ANOVA, analysis of variance; PD-1, programmed cell death protein-1; CTLA-4, cytotoxic T-lymphocyte associated protein-4.

.
